# Integration of flux measurements and pharmacological controls to optimize stable isotope-resolved metabolomics workflows and interpretation

**DOI:** 10.1038/s41598-019-50183-3

**Published:** 2019-09-23

**Authors:** Pawel K. Lorkiewicz, Andrew A. Gibb, Benjamin R. Rood, Liqing He, Yuting Zheng, Brian F. Clem, Xiang Zhang, Bradford G. Hill

**Affiliations:** 1 Department of Medicine, Division of Environmental Medicine, Christina Lee Brown Envirome Institute, Diabetes and Obesity Center, University of Louisville, Louisville, USA; 2 Department of Chemistry, Center for Regulatory and Environmental Analytical Metabolomics, University of Louisville, Louisville, USA; 3 Department of Biochemistry and Molecular Genetics, University of Louisville, Louisville, KY USA; 4 Lewis Katz School of Medicine, Temple University, Philadelphia, PA USA

**Keywords:** Metabolomics, Cell biology, Cardiovascular biology

## Abstract

Stable isotope-resolved metabolomics (SIRM) provides information regarding the relative activity of numerous metabolic pathways and the contribution of nutrients to specific metabolite pools; however, SIRM experiments can be difficult to execute, and data interpretation is challenging. Furthermore, standardization of analytical procedures and workflows remain significant obstacles for widespread reproducibility. Here, we demonstrate the workflow of a typical SIRM experiment and suggest experimental controls and measures of cross-validation that improve data interpretation. Inhibitors of glycolysis and oxidative phosphorylation as well as mitochondrial uncouplers serve as pharmacological controls, which help define metabolic flux configurations that occur under well-controlled metabolic states. We demonstrate how such controls and time course labeling experiments improve confidence in metabolite assignments as well as delineate metabolic pathway relationships. Moreover, we demonstrate how radiolabeled tracers and extracellular flux analyses integrate with SIRM to improve data interpretation. Collectively, these results show how integration of flux methodologies and use of pharmacological controls increase confidence in SIRM data and provide new biological insights.

## Introduction

Metabolism underpins life. Cells take energy from their surroundings in the form of carbon- and nitrogen-containing compounds and return to their surroundings equal amounts of energy as heat and waste products. Although metabolism increases the level of exogenic disorder, it reduces local disorganization, thereby upholding cellular structure or enabling growth or adaptation to changing environmental conditions^1^. This requires joint regulation of catabolic and anabolic pathways, which must be fine-tuned to not only produce ATP but also to synthesize appropriate quantities of metabolites for synthesizing membranes, nucleic acids, and proteins. Although the interrelationships of metabolic pathways are clearly important to understand, it is difficult to assess how numerous pathways change in a biological sample.

Metabolomics evolved to address this issue. Although changes in metabolite levels can provide evidence of altered metabolic pathway activity, snapshot concentrations do not clearly resolve pathway flux. The incorporation of heavy isotope-labeled nutrient tracers, however, empowers metabolomics with the capacity to resolve differences in relative metabolic pathway flux and to assess the contribution of different nutrient sources to catabolic and anabolic pathways. For example, provision of ^13^C-labeled glucose (or other stably labeled nutrients) to cells, tissues, or organisms leads to time-dependent incorporation of ^13^C into metabolic intermediates or biosynthetic endproducts. The metabolite labeling patterns reflect shifts in the mass of metabolites and can be assigned as isotopologues or isotopomers. Assessed from such data are temporal differences in isotope enrichment, changes in the labeling pattern, or differences in the contribution of nutrients to a metabolite pool, which collectively provide intricate knowledge of the state of metabolism in cells or tissues^2,3^.

While useful, such stable isotope-resolved metabolomics (SIRM) experiments are not simple to perform. These experiments require knowledge and expertise in not only biochemistry and metabolism but in technical niches such as nuclear magnetic resonance (NMR), chromatography, and mass spectrometry (MS). In addition, the large amount of acquired data requires specialized software for spectral deconvolution, metabolite assignment, natural abundance removal, isotopologue assignment, normalization, and quantification. This means that expertise ranging from wet lab biology to *in silico* software development may be involved in the acquisition, assembly, and interpretation of stable isotope metabolomics data. In addition, the instrumentation and reagents are expensive, and the experiments *in toto* are time consuming. Therefore, optimization of experimental design and of communication between involved personnel is essential for obtaining valid data and interpreting results.

In this study, we demonstrate the importance of pharmacological controls for increasing confidence in metabolite assignments and data interpretation. The addition of specific inhibitors or activators of metabolic pathways permits cross-validation with other types of flux measurements and helps identify problems concerning metabolite detection or assignment. In particular, use of such controls facilitates constructive communication between technical personnel and biologists as well as assists in the identification of metabolic loci that could contribute to changes in cell phenotype. Metabolic flux configurations achieved using inhibitors or activators of key nodes of metabolism should allow for the development of an atlas of cell-specific metabolite enrichment patterns corresponding to a range of metabolic states, thereby improving data interpretation and increasing reproducibility within and between laboratories.

## Results and Discussion

**Figure 1:**
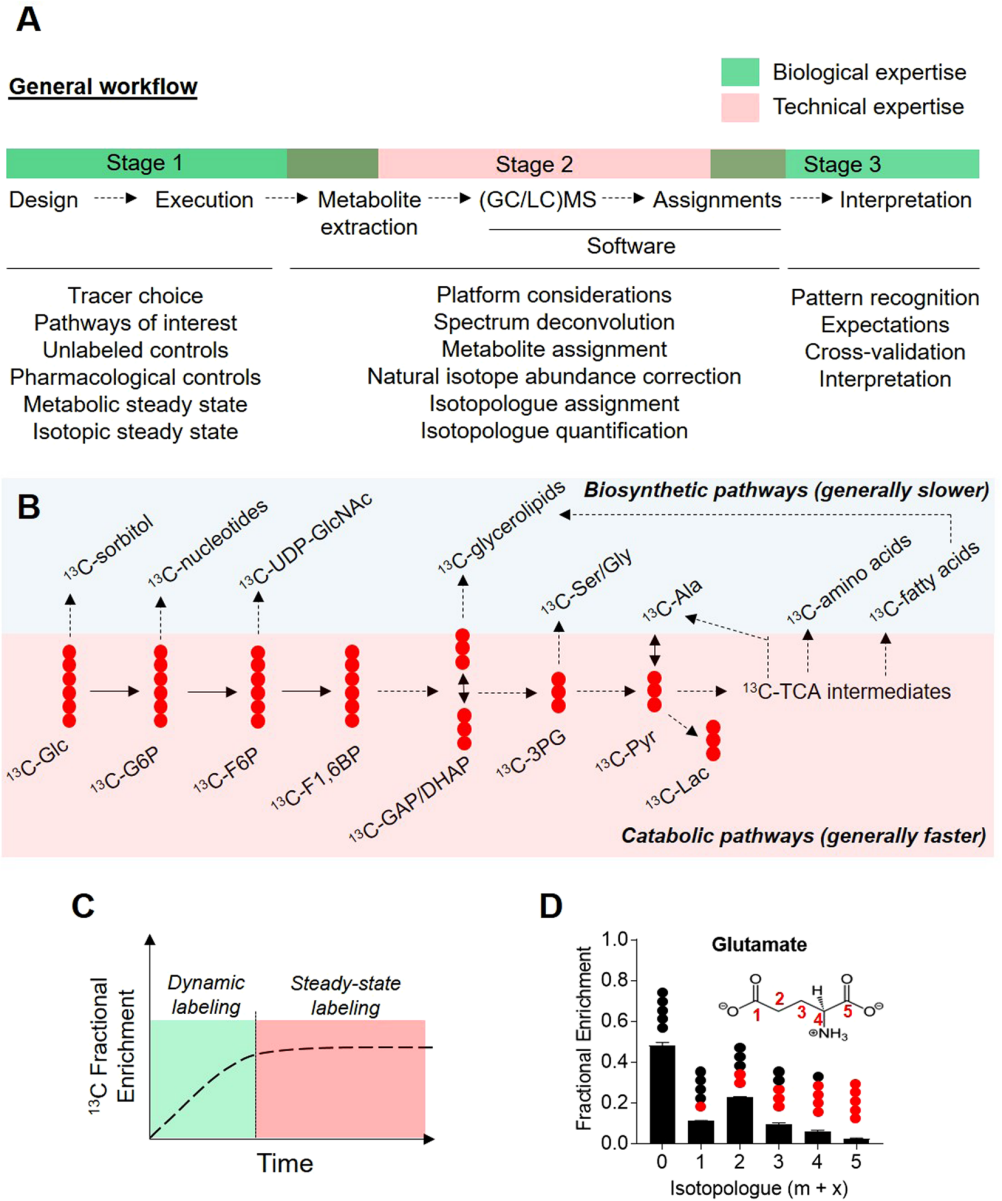
General workflow and considerations for SIRM analyses. (**A**) Schematic illustrating the stages involved and expertise required in a typical SIRM experiment, including considerations at each stage; (**B**) Schematic illustrating assumptions regarding catabolic pathway and anabolic pathway flux; (**C**) Illustration of the dynamic and steady state isotopic labeling phases which must be considered in a typical SIRM experiment; and (**D**) Example of a ^13^C fractional enrichment plot of glutamate. Note that isotopologue analyses do not provide positional information of the isotopic atoms.

Stable isotope metabolomics experiments require knowledge of biology and metabolism, wet lab expertise, and expertise in mass spectrometry and bioinformatics (Fig. 1A). A list of terminology germane to stable isotope metabolomics is found in Table 1. Below, we discuss the major stages of a typical stable isotope metabolomics experiment.

**Table 1:** Terminology germane to stable isotope-resolved metabolomics workflows and interpretation.

Term	Definition
*Absolute flux*	Rate of mass flow in a metabolic pathway. Rates are dependent on the availability of precursor metabolites and on enzymatic turnover. An absolute flux rate is generally described in terms of moles/unit time/unit mass or cell number.
*Anaplerosis*	Series of enzymatic reactions or pathways, excluding glucose oxidation, that replenish pools of metabolic intermediates in the Krebs cycle. Typical anaplerotic pathways include those regulated by pyruvate carboxylase, malic enzyme, transamination reactions between oxaloacetate or malate and their corresponding amino acids (e.g., aspartate, glutamate), formation of succinyl CoA from propionyl CoA precursors (e.g., branched chain amino acids, propionate, odd-carbon ketone bodies), etc.
*Dynamic labeling*	The phase of isotopic labeling in which metabolites are not saturated with isotope label. Measuring fractional enrichment during this phase of labeling delineates how fast a metabolite pool becomes labeled. Dynamic labeling patterns are dependent on the metabolite pool level and the turnover of a given metabolite, which is related to the flux of the metabolic pathway.
*Fractional enrichment*	The fraction of a metabolite pool that is enriched with an isotopic label.
*Glucose oxidation*	The complete catabolism of glucose. Glucose oxidation commences with the breakdown of glucose via glycolysis, which yields pyruvate and is oxidized further in mitochondria to provide energy for the cell.
*Glycolysis*	Metabolic pathway that converts glucose into pyruvate via a series of intermediate steps. Conversion of pyruvate to lactate regenerates NAD^+^ and can allow glycolysis to continue.
*Isobar*	Atomic or molecular species with the same nominal mass as the target analyte. Isobaric compounds may be structural isomers or structurally unrelated compounds with the same nominal mass.
*Isomer*	Compound with the same molecular formula as a target analyte. Isomers have different arrangements of atoms, which can convey different properties.
*Isotopic steady state*	The phase of isotopic labeling in which metabolites are saturated with isotope. Defining the contribution of a substrate to a particular metabolite pool requires that the metabolite pool be in the isotopic steady state phase of labeling.
*Isotopologue*	A molecule that differs in its isotopic composition, with at least one atom having a different number of neutrons than the parent molecule. For example, glutamate with 1, 2, 3, 4, or 5 isotopic atoms (See Fig. 1D).
*Isotopomer*	An isotopomer is an isotopologue containing positional information of the isotopic atoms.
*Krebs Cycle*	A hub of metabolism located within mitochondria that has central importance for both energy production and biosynthesis. Also, called citric acid cycle and tricarboxylic acid cycle.
*Metabolic steady state*	The state or condition of metabolism in which the rates of substrate uptake and utilization are the same over time. Under conditions of metabolic steady state, the metabolite levels remain constant.
*Metabolite pool*	The reservoir of a metabolite upon which enzymes can operate. Metabolite pools may be compartmentalized in the cell (e.g., in the cytosol and/or mitochondrion); however, the total pool is commonly extracted for SIRM analysis.
*Metabolic pseudo-steady state*	Assumption that the state of metabolism does not change under a given set of conditions.
*Natural abundance*	The abundance of isotopes of an element that are naturally found on a planet.
*Relative flux*	Relative activity of a metabolic pathway in experimental conditions compared with control conditions. The degree of isotopic enrichment in a metabolite, and the pattern of labeling, can be used to infer higher or lower metabolic pathway activity as long as the analysis is performed in the dynamic labeling phase.
*Scrambling*	Scrambling describes a hybrid metabolite labeling pattern resulting from a convergence of multiple pathways or several turns of a metabolic cycle (e.g., Krebs cycle). This complex isotopic labeling can obfuscate interpretation of SIRM data.
*Stable isotope resolved metabolomics*	An approach that uses nuclear magnetic resonance and/or mass spectrometry to determine the fate of individual atoms derived from stable isotope-enriched precursors (e.g., ^13^C-glucose) in biological systems. By measuring the fractional enrichment of stably labeled metabolic intermediates and endproducts, it is used to deduce metabolic pathway and network activity.

### Stage 1 – Experimental design and execution

Biological expertise and knowledge of biochemistry are required for designing and executing SIRM experiments. This involves identifying tracer(s) relevant to the pathway(s) of interest and planning experiments to derive clean results, the latter of which should strive to minimize factors that obfuscate interpretation. Considerations for metabolic steady state and the time required for isotope enrichment are particularly important.

A metabolic steady state is ideal for stable isotope tracing. Under conditions of a metabolic steady state, the intracellular metabolite concentrations and metabolic fluxes of a cell population are constant. Because progression of the cell cycle is coordinated with bioenergetic and biosynthetic demands^4–7^, differences in cell proliferation will affect substrate uptake and utilization, and thus metabolite labeling. The exponential phase of growth is commonly assumed to reflect a near steady state condition, if nutrient supply is maintained^2,8^. For controlling nutrient supply, a bioreactor (commonly called a nutrostrat) which adds fresh medium and removes waste products ensures that the extracellular environment is optimized for cell growth^9,10^. Often, experiments are performed at a metabolic pseudo-steady state, in which deviations in extracellular nutrient concentration and intracellular metabolite concentrations and fluxes are considered negligible over the course of the experiment. Many cell culture media compositions contain supraphysiologic substrate levels to prevent nutrient depletion over the course of culture. For example, incorporation of ^13^C from ^13^C_6_-glucose into glycolytic intermediates is expected to occur within minutes in most cell types, whereas tricarboxylic acid cycle (TCA) cycle intermediates may require a few hours for sufficient ^13^C accumulation. Some biosynthetic pathway-derived intermediates and end products require longer time frames for sufficient label incorporation (Fig. 1B). Thus, at physiological concentrations, glucose would not become limiting if only ^13^C enrichment in glycolytic intermediates are measured; however, higher concentrations of glucose may be required to prevent nutrient depletion for assessment of label incorporation into TCA cycle or biosynthetic pathway metabolites.

After provision of an isotopically labeled source, metabolite isotope incorporation will increase in a time-dependent manner. This phase of isotope labeling is referred to as dynamic labeling. After ^13^C incorporation in a metabolite pool is saturated, isotopic steady state labeling is achieved (Fig. 1C). Importantly, the time required to achieve isotopic steady state is both metabolite- and tracer-dependent and is assessed empirically. Dynamic and steady state labeling are discussed in detail below.

### Stage 2 – Sample preparation, data acquisition, and data analysis

Proper sample preparation and data analysis are critical for reliable interpretations of SIRM experimental results. In this section, we discuss sample preparation, metabolite separation and detection by liquid chromatography mass spectrometry (LC-MS), and data processing and analysis.

#### Sample preparation

Rapid enzymatic inactivation and efficient metabolite extraction are crucial steps to provide a snap shot of isotopologue labeling. Collection vessels and solvents should be of the highest purity to prevent contamination, especially by typical metabolite contaminants such as lactate^11^ and palmitate^12^. The most effective solvents for quenching are cold (–20 °C) acetonitrile or ultracold (−80 °C) methanol^13,14^. While methanol is sufficient to quench and extract a broad range of polar and some nonpolar metabolites^15^, maximal extraction of polar metabolites—including central carbon intermediates, amino acids and dipeptides, nucleosides and nucleotides, short-chain fatty acids, and some mono- and di-glycerides—can be achieved using 50–60% acetonitrile (v/v; acetonitrile/water)^13,14^; for the empirical results presented below, a 60% acetonitrile solution was used. Extraction of more hydrophobic species and lipids may require solvents such as chloroform^16^ or methyl tert-butyl ether^17^.

After sample collection, the extracts are processed and centrifuged at ≤4 °C, and the supernatants may be stored at –80 °C. Frequently, the solvent volume is reduced or removed and replaced with a solvent suitable for metabolite detection. Volatile solvents (e.g., methanol, chloroform) are removed using a stream of nitrogen gas; other solvents such as acetonitrile/water can be removed with a freeze dryer. Vacuum concentrators and centrifugal dryers may contribute to sample loss, contamination, and metabolite degradation (due to overheating) and therefore are not recommended.

#### Platform considerations

While many platforms are suitable for SIRM, MS-based workflows are common in most laboratories due to their capacity for broad metabolite coverage, high sensitivity, and the availability of software packages for data analysis. Liquid chromatography prior to MS analysis reduces spectral complexity and minimizes spectral overlap between labeled species by separating some species of identical molecular mass (e.g., isobars and isomers)^3^. Pairing LC with high resolution, accurate mass (HRAM) spectrometers such as orbitraps or time-of-flight instruments empowers the LC-MS approach with the ability to perform SIRM in an untargeted manner. Knowledge of metabolite retention time, accurate m/z, MS/MS spectrum, and use of authentic standards improves peak assignment accuracy. Other techniques, such as gas chromatography mass spectrometry (GC-MS)^18–21^ and capillary electrophoresis MS (CE-MS)^22^, can be used in targeted and non-targeted applications, and unit resolution triple quadrupole-based LC-MS is effective for targeted metabolomics methods^23,24^.

#### Spectrum deconvolution and metabolite assignment

Mass spectra generated for stable isotope-labeled metabolites differ significantly from those of unlabeled samples. Incorporation of heavy atoms (e.g. ^13^C) from the stable isotope tracer increases the complexity of the mass spectrum and requires additional analytic steps. Several bioinformatics tools have been developed to analyze SIRM data, e.g., see^25–30^. Our group has used the following workflows for peak assignment: Premise^31,32^, MetSign^33^, and El-Maven/Polly Phi^TM^^34,35^; the last two of these software packages can facilitate large metabolomics databases or custom target lists. A key advantage of the El-Maven/Polly Phi^TM^ software is that it automatically calculates all possible isotopologue m/z ratios for heavy atoms specified by the user (e.g. ^13^C, ^15^N, etc.) and performs peak matching and assignments based on its algorithm and a set of user modifiable criteria. Metabolite peaks are assigned and confirmed using accurate m/z, retention time, and the MS/MS spectrum performed on peaks from unlabeled sample controls. These unlabeled controls are biological equivalent samples generated in the same experimental settings but without incubation with the labeled tracer. Unlabeled samples are important because: (1) they help define the sample’s metabolome composition; (2) by absence of tracer atoms, they help validate isotopologue assignments; and (3) they can provide the relative concentration for a given metabolite.

#### Natural abundance correction

After spectral deconvolution and assignment, the contribution of naturally occurring heavy isotopes (e.g. ^2^H is 0.0115%, ^13^C is 1.07% and ^15^N is 0.368^36^) is removed, yielding corrected peak intensities. By removing the natural abundance from spectral data, overestimation of fractional enrichment can be avoided. Natural abundance correction algorithms are a part of several software pipelines. For example, we have used online platforms based on a script described by Moseley *et al*.^37^ as well as MetSign^33^. Here, we use the IsoCorrect module—part of a larger Elucidata PollyPhi™ Platform^34^. A more detailed discussion of natural abundance correction can be found in the following reference^21^.

#### Data analysis and verification

Proper vetting of the assignments is essential to exclude inaccuracies and avoid incorrect data interpretation. The steps involved for data inspection require knowledge of analytical chemistry and metabolism. Data validation starts by setting up *a priori* criteria to maintain coherence within the dataset and eliminate possible user bias. The granularity of these criteria varies between experiments, and commonly encountered problems are summarized in Table 2. In general, replicate data are manually inspected for assignment errors and omissions. This allows the user to identify and address software-related issues quickly. Nevertheless, reexamination of chromatograms and mass spectra may be necessary. This is especially true when there is high intra-replicate variability, which is commonly caused by poor detection of low abundance metabolites. For poorly detected metabolites, increasing the starting material may be sufficient to obtain interpretable results.

**Table 2:** Common problems encountered in SIRM.

Problem	Possible reason	Actions taken
*No labeling in one or more samples (only monoisotopic peak present) in a metabolite that should be labeled*	Signal intensity of isotopologue peaks below detection limit or software- determined threshold Insufficient time for labeling	Inspect original spectra or absolute intensities in raw data format. If abundances are near the noise level, exclude assignment. If isotopologue signals that match retention time and mass are clearly present but not detected, manually assign peaks or re-adjust limit of detection and repeat software analyses. Perform time course labeling studies.
*Metabolite not detected in one or more replicate samples*	Metabolite concentration too low to reach limit-of-detection, or m/z and/or retention time shifted over the set threshold	Examine chromatogram, and original mass spectra. If metabolite is missing, exclude sample. Large drifts signify possible issues with instrument. Perform system maintenance, tuning and calibration. Reacquire and reprocess spectrum.
*Enrichment markedly different from other replicate samples*	Spectral overlap of one or more isotopologue peaks and co-eluting isobaric species Experimental variation due to user error Signal artifact	Examine chromatogram, and original mass spectra. If peaks missing are near the noise level, exclude assignment. Inspect unlabeled mass spectrum for possible contamination and “ghost” isotopologue peaks. If present, and cannot be eliminated, exclude assignment. Consult statistician to determine if the sample qualifies as an outlier. Exclude sample, only if justified. Check mass spectrum manually. Observe baselines and inspect any possible spikes in signal/noise. If issues found, exclude entire sample. Distortions present in multiple samples can suggest instrument issues. Perform system maintenance, tuning and calibration. Reacquire and reprocess spectrum.

Supplementary Fig. 1 illustrates the differences in chromatograms and raw mass spectra of citrate and α-ketoglutarate. High signal-to-noise chromatographic peaks were deconvoluted and assigned as citrate (Supplementary Fig. 1A). The corresponding mass spectrum (Supplementary Fig. 1C) revealed high abundance isotopologue peaks and undistorted labeling. In contrast, in this experiment, α-ketoglutarate signals were difficult to distinguish from instrument noise, increasing the likelihood for misassignment (Supplementary Fig. 1B). The MS spectrum of α-ketoglutarate showed that while the two most abundant isotopologues (m + 0 and m + 5) were present, others were undetected (Supplementary Fig. 1D). When peaks are below the software threshold of detection, metabolite peaks can be manually inspected, and assignments can be entered based on accurate retention times and masses. Removal of outliers is generally discouraged unless deemed statistically appropriate based on *a priori* criteria in the study design. For replicates with several poorly detected metabolites, the analyst may choose to reacquire the spectra or repeat the entire labeling experiment using more biological starting material.

Apart from analytical knowledge, expertise in metabolism, the integration of additional metabolic flux measurements, and the use of pharmacological controls may uncover additional data integrity issues. These frequently involve a mismatch in labeling patterns between a suspected metabolite and other pathway constituents. Such inconsistencies prompt additional steps to reexamine and evaluate assigned data. Furthermore, pharmacological controls that target critical steps in metabolic pathways can identify possible assignment issues, especially if the labeling patterns do not agree with textbook biochemistry. Finally, heavy isotope enrichment found in unlabeled samples that exceeds natural abundance suggests misassignments due to peak overlaps and requires intervention. Once assignments are corrected and possible outliers (e.g., undetected metabolites) are removed, they can be plotted and interpreted.

### Stage 3 – Data interpretation

After metabolites and isotopologues are assigned and quantified, fractional enrichment data are plotted for interpretation. An example of a fractional enrichment plot is shown in Fig. 1D. Here, ^13^C enrichment into glutamate is shown for cells provided with ^13^C_6_-glucose. The degree of ^13^C incorporation is indicated by the fractional (isotopic) enrichment values for each isotopologue (m + x). As mentioned earlier, the m + 0 isotopologue indicates the fraction of unlabeled glutamate, whereas each successive isotopologue shows the relative abundance of ^13^C incorporated into 1, 2, 3, 4, or all 5 carbons of glutamate. For most LC-MS approaches, it is not possible to ascertain positional information of the labeled atom (i.e., distinguish isotopomers); however, strategic fragmentation and mathematical modeling strategies can provide position-specific enrichment information^38^.

Another way to examine metabolite labeling is by adding all isotopologues together, which provides the total isotopic enrichment (i.e. % metabolite enrichment) for a given metabolite pool. Importantly, when a time-course experiment is performed, plotting the isotopic enrichment is the only means by which dynamic and isotopic steady state may be distinguished. Shown in Supplementary Fig. 2 is a sampling of time-dependent isotopic enrichment in metabolites from several metabolic pathways from neonatal rat cardiomyocytes (NRCMs) incubated with ^13^C_6_-glucose. Differences in pathway flux are readily appreciated by comparing enrichment in metabolites derived from slower pathways, such as the pentose phosphate pathway, versus metabolites in faster pathways, such as glycolysis. For example, 3′AMP and glutathione are in the dynamic labeling phase throughout the time course, whereas the ^13^C label saturates the 3-phosphoglycerate (3PG) pool by at least 4 h. Metabolites associated with the Krebs cycle demonstrate intermediate labeling velocities, with the dynamic labeling phase occurring within 0–12 h of incubation with ^13^C_6_-glucose.

Relative flux differences in metabolic pathways may be inferred from incorporation of isotopic tracer into metabolites during the dynamic labeling phase. Resolving changes in relative metabolic pathway flux can be assessed by direct interpretation of stable isotope labeling patterns^2^, without the need for formal ^13^C flux analysis, which utilizes mathematical modeling^39–41^. For example, NRCMs incubated with ^13^C_6_-glucose show purine (3′AMP) labeling in the dynamic phase through 18 h. Therefore, a hypothetical decrease in m+0 fractional enrichment and an increase in m + 5 labeling in purines in an experimental group within this timeframe would suggest higher rates of ribose incorporation and increased pathway flux.

The contribution of a tracer to a metabolite pool should be assessed after isotopic enrichment has reached steady state. For example, ^13^C labeling in citrate saturates by 10–12 h of introducing ^13^C_6_-glucose to NRCMs. Enrichment approaches 90% by 18 h, suggesting that glucose-derived carbon is the predominant source of carbon for citrate synthesis (Supplementary Fig. 2).

**Figure 2:**
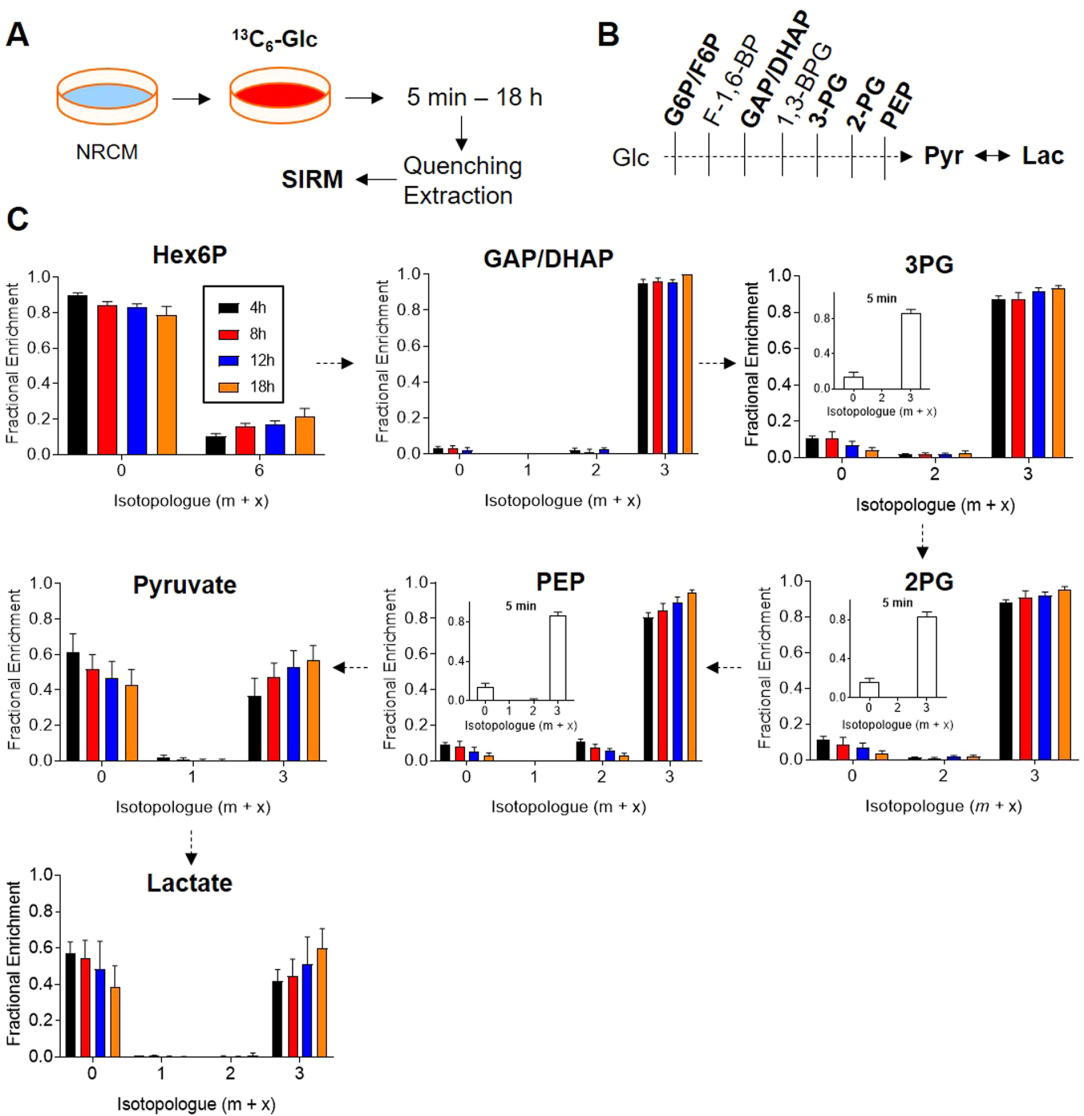
Time course of ^13^C enrichment in glycolytic intermediates. Neonatal rat cardiac myocytes (NRCMs) were cultured in medium containing ^13^C_6_-glucose for 5 min, 4 h, 8 h, 12 h, or 18 h, followed by quenching, metabolite extraction, and SIRM analyses: (**A**) Schematic of experimental protocol; (**B**) Condensed schematic of glycolysis; note that the metabolites in bold are shown in these analyses; and (**C**) Fractional enrichment of ^13^C into glycolytic intermediates. Insets are data derived from a separate experiment in which ^13^C_6_-glucose was provided for 5 min. n = 6 replicates per group, pooled from NRCMs isolated from three independent litters.

Analysis of glycolysis: Although fractional enrichment plots of glycolytic intermediates can resolve changes in glycolysis, examining relative changes in glycolytic flux can be difficult. In NRCMs, glycolytic intermediates such as the glyceraldehyde 3-phosphate/dihydroxyacetone phosphate pool (GAP/DHAP), 3-phosphoglycerate (3PG), 2-phosphoglycerate (2PG), and phosphoenolpyruvate (PEP) show nearly complete saturation with label by 4 h (Fig. 2A–C). In another experiment, NRCMs were incubated with medium containing ^13^C_6_-glucose for 5 min (insets). Results from this experiment suggest that isotopic steady state is reached within minutes of ^13^C_6_-glucose provision. Such rapid attainment of isotopic steady state makes the assessment of glycolytic flux by isotopic labeling difficult. In such cases, glycolytic rates can be measured by assessing the levels of ^13^C_3_-lactate and ^13^C_6_-glucose in the incubation medium^42^ as well as by orthogonal methods, such as methods based on radiolabeled tracers ([5-^3^H]-glucose) or extracellular flux analysis.

That 40–60% of the pyruvate and lactate pools were unlabeled suggests dilution by non-labeled substrates and is supported by the presence of unlabeled pyruvate in the medium. The presence of primarily the m + 3 isotopologue in these intermediates indicates that only ^13^C_6_-labeled glucose was used for glycolysis. Similarly, the hexose 6-phosphate pool consisted of only m + 6 labeling, which indicates low levels of fructose bisphosphatase activity in NRCMs. The presence of other isotopologues, e.g., m + 3 labeling, in hexose 6-phosphate in cells incubated with only ^13^C_6_-glucose could indicate gluconeogenic activity, but this isotopologue was not observed in these studies. Interestingly, in NRCMs, the hexose 6-phosphate pool had relatively modest labeling at the m + 6 isotopologue, which indicates unlabeled isomers of hexose 6-phosphate (Fig. 2C).

**Figure 3:**
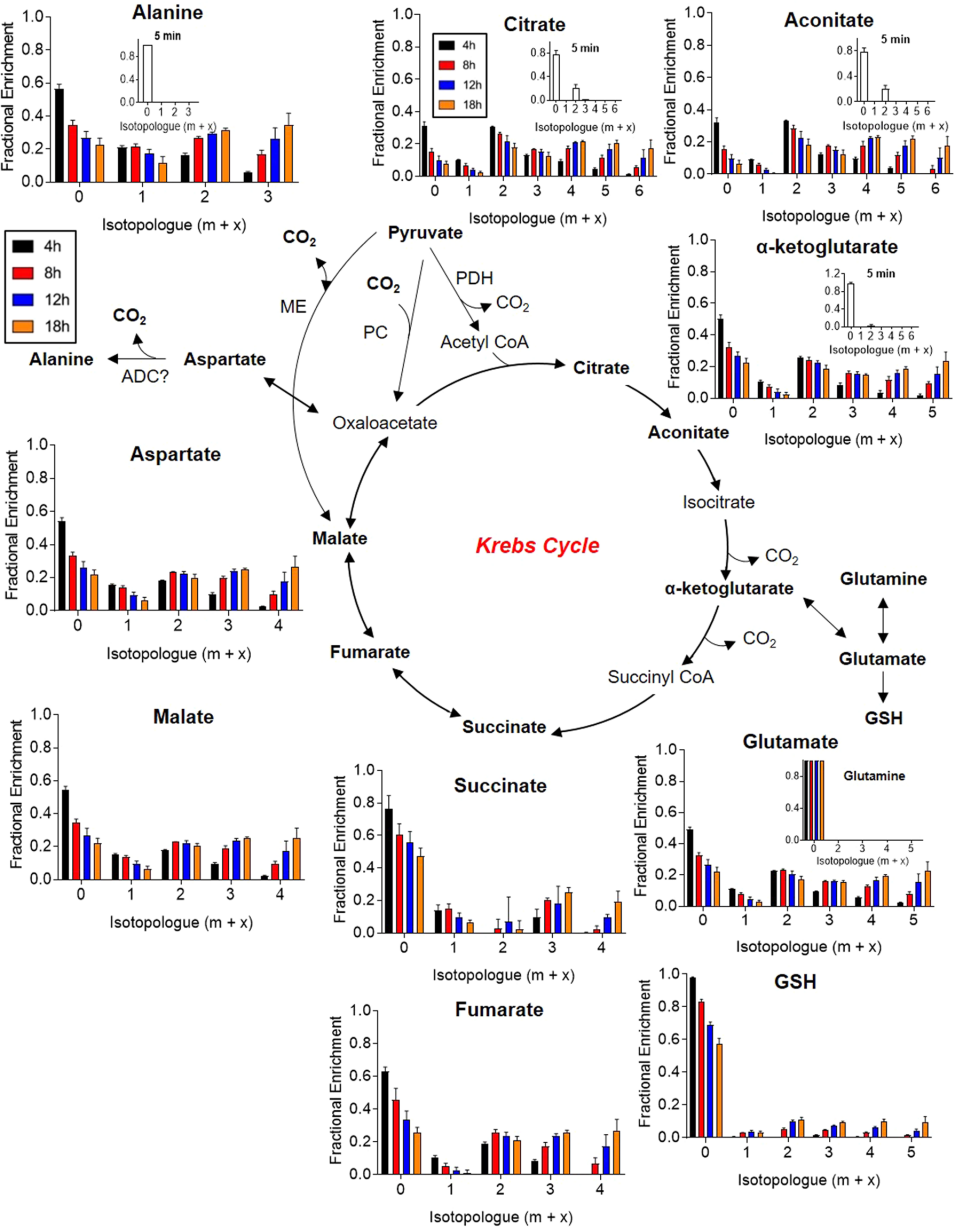
Time course of ^13^C enrichment in Krebs cycle intermediates. Neonatal rat cardiac myocytes (NRCMs) were cultured in medium containing ^13^C_6_-glucose for 5 min, 4 h, 8 h, 12 h, or 18 h, followed by quenching, metabolite extraction, and SIRM analyses: (**A**) Fractional enrichment of ^13^C into Krebs cycle intermediates. Insets are data derived from a separate experiment in which ^13^C_6_-glucose was provided for 5 min. n = 4–6 replicates per group, pooled from NRCMs isolated from three independent litters. PDH, pyruvate dehydrogenase; ME, malic enzyme; PC, pyruvate carboxylase; ADC, aspartate decarboxylase.

Analysis of Krebs cycle activity: As expected, Krebs cycle intermediates in NRCMs demonstrate time-dependent ^13^C labeling (Fig. 3), from which several conclusions may be drawn. The labeling patterns in Krebs cycle intermediates are suggestive of both pyruvate dehydrogenase-mediated and anaplerotic incorporation of the ^13^C atoms. Enrichment in m + 2 isotopologues indicate the first turn of the Krebs cycle (i.e. m + 2). The m + 4, m + 5, and m + 6 isotopologue labeling in citrate and aconitate may indicate the 2^nd^ and 3^rd^ turns of the Krebs cycle, with label saturation apparent in the m + 4 isotopologues by 12 h. Labeling the cells with ^13^C_6_-glucose for 5 min led to approximately 20% ^13^C enrichment (m + 2) in citrate and aconitate; however, little to no labeling was found in α-ketoglutarate (Fig. 3, insets) or any other Krebs cycle metabolite (data not shown). These data suggest that incorporation of label into citrate is faster than that through “downstream” metabolites, which is consistent with relatively higher metabolic needs for citrate due to fatty acid synthesis.

Product-precursor relationships are also important to consider. For example, by 4 h, >70% of the citrate pool was labeled (at m + 1, m + 2, m + 3, m + 4, or m + 5), yet pyruvate (see Fig. 2C) showed only 40% labeling (at m + 3). This could indicate the presence of more than one pool of pyruvate, with the labeled pyruvate pool contributing more to the Krebs cycle than the unlabeled pool; however, the Krebs cycle carries large flux, and given that the levels of unlabeled pyruvate in the medium would progressively decrease over time, earlier times of labeling may be informative. Indeed, a 5 min labeling study revealed lower fractional enrichment in citrate than in pyruvate (Supplementary Fig. 3), which refutes the idea that two pools of pyruvate contribute to citrate formation in NRCMs. These data exemplify the importance of time course data for delineating product-precursor relationships and for interpreting metabolic pathway activities.

The fact that glutamate and glutathione (GSH) were labeled suggests that NRCMs use glucose as a carbon source for glutathione synthesis. Glutamine was not labeled, which is expected given that unlabeled glutamine is provided in relatively high amounts in the culture medium (Fig. 3). The m + 3 labeling, e.g., in ^13^C_3_-malate and -fumarate, may also indicate anaplerotic activity^43,44^, likely through either pyruvate carboxylase^44^ or malic enzyme^45^. The kinetics of labeling in these isotopologues, when compared to that of the PDH-supported labeling (m + 2), indicate that these carboxylation reactions are generally slower than pyruvate decarboxylation reactions in NRCMs. Indeed, 5 min of culture of NRCMs with ^13^C_6_-glucose led to labeling of citrate at only m + 2 and did not label malate (Supplementary Fig. 3C).

While the labeling patterns of Krebs cycle intermediates and associated metabolites (e.g., aspartate, glutamate) are generally consistent, questions arise with regard to succinate and alanine. Succinate was almost completely devoid of ^13^C incorporation derived from PDH-mediated Krebs cycle activity. Nevertheless, the fact that independent studies [e.g.^44^,] show relatively low levels of ^13^C incorporation into succinate compared with other Krebs cycle intermediates suggest that these data may be valid; they might be explained by different intracellular pools of succinate, in which a relatively minor succinate pool is used for fumarate production. Surprisingly, alanine labeling is most similar to that of malate and aspartate (Fig. 3) and does not follow that of pyruvate (see Fig. 2C). This would suggest that, in NRCMs, alanine does not arise by alanine transamination of pyruvate but rather via aspartate decarboxylase. Conclusive evidence for this mechanism could be provided by interventions that target aspartate decarboxylase directly.

Importance of complementary metabolic assays and pharmacological controls: The preceding data illustrate how isotopologue pattern matching and time course information can impart confidence or skepticism in stable isotope metabolomics data as well as give rise to new, interesting questions. While technical controls—including proper retention time and mass values according to metabolite standards—provide confidence in metabolite assignment and isotopologue quantification, the metabolome consists of many similar metabolites that may have nearly identical masses and may be difficult to resolve by chromatography. While some newer technologies (e.g., differential mobility spectrometry) can address some of these challenges^38,46^, additional methods to assess the veracity of stable isotope metabolomics data and to standardize workflows within and between laboratories could increase the reproducibility, certainty, and usefulness of SIRM results.

**Figure 4:**
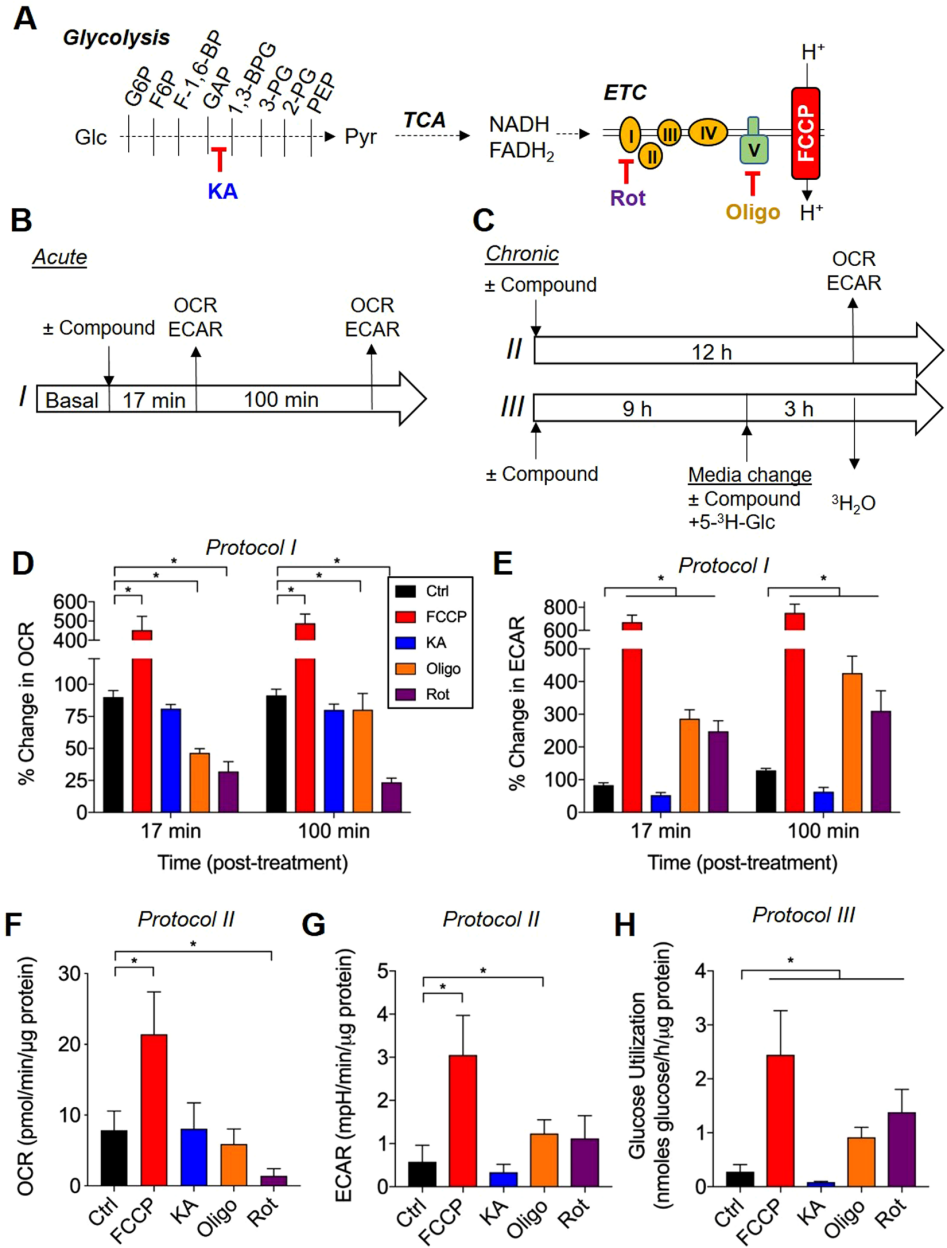
Mitochondrial and glycolytic activity under controlled metabolic states. Extracellular flux analyses and radiolabeled glucose utilization assays in NRCMs treated with metabolic inhibitors or activators: (**A**) Schematic illustrating the inhibitors used: KA, koningic acid (10 µM); Rot, rotenone (1 µM); Oligo, oligomycin (1 µM); FCCP, carbonyl cyanide 4-(trifluoromethoxy)phenylhydrazone (1 µM); (**B**) Schematic of the protocol for measuring acute changes in mitochondrial and glycolytic activity with each pharmacological compound; (**C**) Schematic of the protocol for measuring chronic changes in mitochondrial and glycolytic activity with each pharmacological compound; (**D**) Oxygen consumption rate (OCR) after incubation with each compound for 17 and 100 min; (**E**) Extracellular acidification rate (ECAR) after incubation with each compound for 17 and 100 min; (**F**) OCR after incubation with each compound for 12 h; (**G**) ECAR after incubation with each compound for 12 h; (**H**) Glucose utilization with each compound, measured using the [5-^3^H]-glucose assay (see Protocol III, panel C). n = 4 independent isolations per group. Data from XF analyses were log-transformed prior to statistical analysis. *p < 0.05.

We gain additional confidence in SIRM data by including pharmacological controls in experimental design. By predicting ^13^C labeling based on the metabolic steps and pathways known to be affected by pharmacological compounds, these experimental groups could be used as positive or negative controls. To examine this, we treated NRCMs with koningic acid (KA)—a known glyceraldehyde 3-phosphate dehydrogenase inhibitor; rotenone—a commonly used inhibitor of mitochondrial Complex I; oligomycin—a commonly used inhibitor of Complex V; or FCCP—an uncoupler that stimulates mitochondrial oxygen consumption (Fig. 4A). We measured mitochondria and glycolytic activity with acute (17 or 100 min: Protocol I) and chronic (12 h: Protocol II, III) treatments with each compound (Fig. 4B,C). As expected, both acute and chronic treatment with FCCP increased oxygen consumption rate (OCR); rotenone decreased OCR both acutely and chronically (Fig. 4D,F). Oligomycin initially decreased OCR by 50%, which is consistent with our previous studies (Fig. 4D)^42,47,48^; however, upon sustained (12 h) exposure, OCR rebounded to baseline levels (Fig. 4F). This is expected given the propensity of oligomycin to increase Δp and thereby promote proton leakage or electron slippage^49^. While KA had no effect on OCR (Fig. 4D,F), KA acutely decreased the extracellular acidification rate (ECAR), whereas oligomycin and rotenone increased ECAR (Fig. 4E). Radiolabeled tracing experiments using [5-^3^H]-glucose (Protocol III; Fig. 4C) provided absolute flux measurements and demonstrated sustained changes in glucose utilization caused by each pharmacological compound (Fig. 4H). Importantly, the pharmacological agents did not affect protein content significantly (Supplementary Fig. 4), which suggests minimal changes in cell viability.

**Figure 5:**
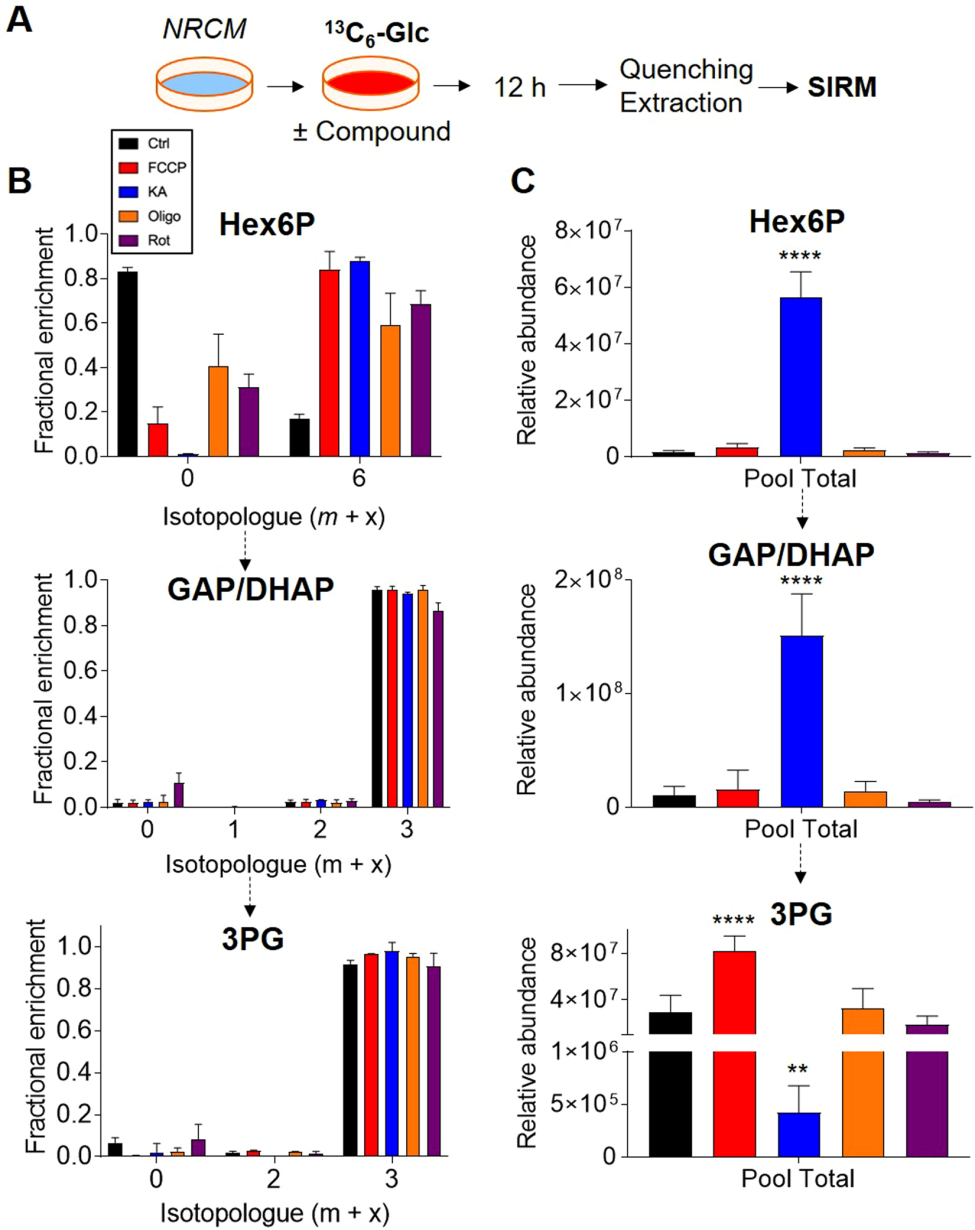
^13^C enrichment in glycolytic intermediates under controlled metabolic states. (**A**) NRCMs were treated with the indicated inhibitor (as in Fig. 4) for 12 h in medium containing 25 mM ^13^C_6_-glucose. Intracellular metabolites were then extracted and subjected to LC/MS for SIRM analysis; (**B**) Fractional enrichment plots of glycolytic intermediates; and (**C**) Relative pool size for indicated glycolytic intermediates. n = 3–6 replicates per group, pooled from NRCMs isolated from three independent litters. **p < 0.01, ****p < 0.0001 vs. Ctrl.

We next examined ^13^C metabolite labeling in NRCMs cultivated with ^13^C_6_-glucose for 12 h in the absence or presence of each pharmacological compound (Fig. 5A). As shown in Fig. 5B, compounds that increased glycolytic activity (i.e., FCCP, oligomycin, and rotenone) robustly increased the fractional enrichment of ^13^C-glucose-derived carbon into hexose 6-phosphate. That KA also augmented ^13^C enrichment into the hexose phosphate pool and that all glycolytic intermediates indicate nearly maximal ^13^C enrichment appear at first inconsistent; however, analysis of relative pool size, knowledge of biochemistry, and additional experiments can provide an explanation. Because KA inhibits GAPDH, crossover theorem^50–53^ predicts that the concentrations of metabolites should increase before the inhibition site and decrease after the inhibition site. Indeed, hexose 6-phosphate and GAP/DHAP levels were remarkably elevated, and levels of metabolites after the GAPDH reaction (e.g., 3PG) were diminished (Fig. 5C). Moreover, because high levels of G6P promote glycogen synthesis^54^ and inhibit glycogen breakdown^55^, the high level of ^13^C enrichment that occurs in the hexose 6-phosphate pool with KA treatment is likely due to diminished G6P dilution by the unlabeled glycogen pool.

In separate experiments, we also evaluated ^3^H-2-deoxyglucose (2DG) uptake, which provides a measure of glucose uptake, in NRCMs treated with KA and FCCP; treatment of NRCMs with unlabeled 2DG (100 mM) was used as a control. As predicted from the labeling patterns of hexose 6-phosphate in Fig. 5, FCCP increased glucose uptake in NRCMs; however, KA did not affect glucose uptake (Supplementary Fig. 5), which suggests that in KA-treated cells glucose-derived intermediates upstream of the GAPDH reaction are likely shunted to ancillary pathways. Such complementary assays help to better define changes in metabolism, especially when the metabolites of interest are assessed at the isotopic steady state phase of labeling.

**Figure 6:**
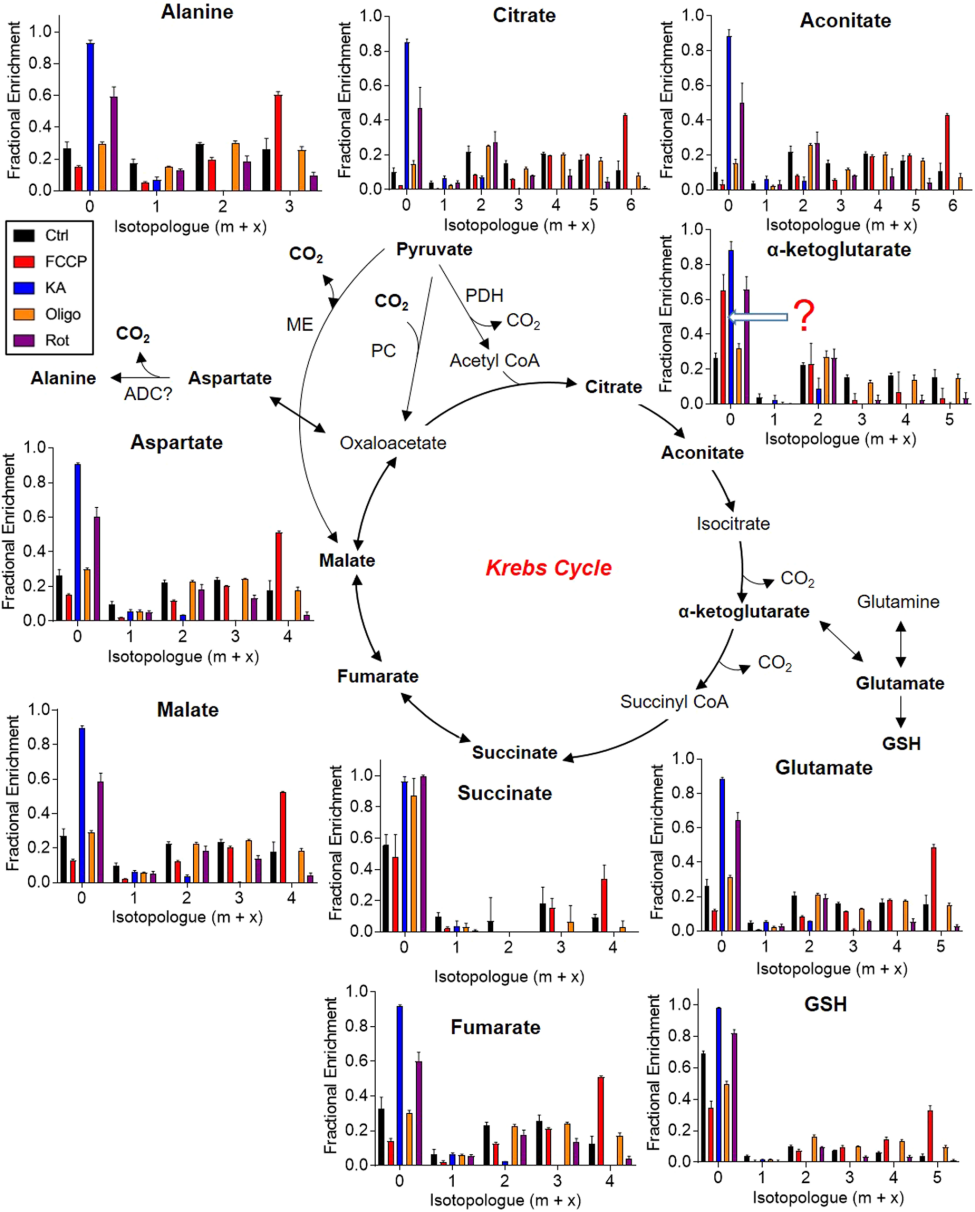
^13^C enrichment in Krebs intermediates under controlled metabolic states. Fractional enrichment plots of Krebs cycle intermediates: NRCMs were treated with the indicated inhibitor (as in Fig. 4) for 12 h in medium containing 25 mM ^13^C_6_-glucose. Intracellular metabolites were then extracted and subjected to LC/MS for SIRM analysis. n = 4–6 replicates per group, pooled from NRCMs isolated from three independent litters. PDH, pyruvate dehydrogenase; ME, malic enzyme; PC, pyruvate carboxylase; ADC, aspartate decarboxylase.

Analysis of ^13^C incorporation into Krebs cycle metabolites provides information on mitochondrial activity. As expected from the mitochondrial OCR measurements (see Fig. 4), FCCP led to higher ^13^C incorporation into Krebs cycle intermediates, with the exception of α-ketoglutarate, which showed less ^13^C incorporation with FCCP (Fig. 6A). Reanalysis of the α-ketoglutarate spectral data indicated a poor signal-to-noise ratio (Supplementary Fig. 1), which diminishes confidence in its adequate detection in this set of experiments. The markedly higher fractional enrichment of the m + 6 isotopologues of citrate and aconitate and the m + 4 isotopologues of succinate, fumarate, and malate could indicate more turns of the Krebs cycle; however, it is important to consider other explanations as well. Longer periods of labeling can increase the possibility of tracer scrambling, which can occur after the tracer has been extensively recycled, or promote more isotope labeling due to less dilution by the unlabeled pool of metabolite precursors. Although increased m + 6 and m + 4 labeling could suggest more turns of the Krebs cycle, it is possible that the same number of turns are occurring with less input from other carbon-donating sources or that there is more input from ^13^C-labeled metabolic products (e.g., ^13^C_3_-lactate or labeled non-essential amino acids); however, integration of pharmacological controls in labeling studies and OCR measurements help resolve these issues. For example, in FCCP-treated NRCMs, complementary data showing higher ^13^C enrichment (Fig. 6) and lower pool size of several Krebs cycle intermediates (Supplementary Fig. 6) as well as higher OCR (Fig. 4F) confirms higher Krebs cycle metabolite turnover and flux.

FCCP also increased ^13^C incorporation into glutathione (GSH) and alanine, indicating that mitochondrial uncoupling also increases the biosynthetic rates of GSH and alanine. The observation that chronic oligomycin treatment did not markedly affect ^13^C labeling of Krebs cycle intermediates (Fig. 6) is not surprising, as OCR was unaffected after 12 h of incubation. As expected based on the OCR data, the Complex I inhibitor rotenone diminished fractional enrichment in all Krebs cycle intermediates as well as in GSH and alanine (Fig. 6A). Collectively, these data illustrate the fractional enrichment patterns that occur in uncoupled mitochondria and when complex I is inhibited.

The glycolysis inhibitor, KA, remarkably diminished ^13^C incorporation into Krebs cycle intermediates, indicating that it decreases glucose oxidation in NRCMs (Fig. 6A). That ^13^C_6_-glucose-derived carbon labeled approximately 80% of the Krebs cycle intermediate pool (e.g., see citrate in Supplementary Fig. 2) indicates that glucose is a primary nutrient for mitochondrial oxidation in cultured NRCMs; that KA generally diminished the levels of Krebs cycle intermediates (see Supplementary Fig. 6) further supports the concept that cultured NRCMs rely on glucose for mitochondrial respiration. Interestingly, mitochondrial respiration data indicate that KA does not markedly inhibit basal OCR (Fig. 4), which suggests inherent flexibility in recruiting other nutrients (e.g., unlabeled pyruvate) to support mitochondrial respiration when glucose is limiting or glucose catabolism is inhibited.

**Figure 7:**
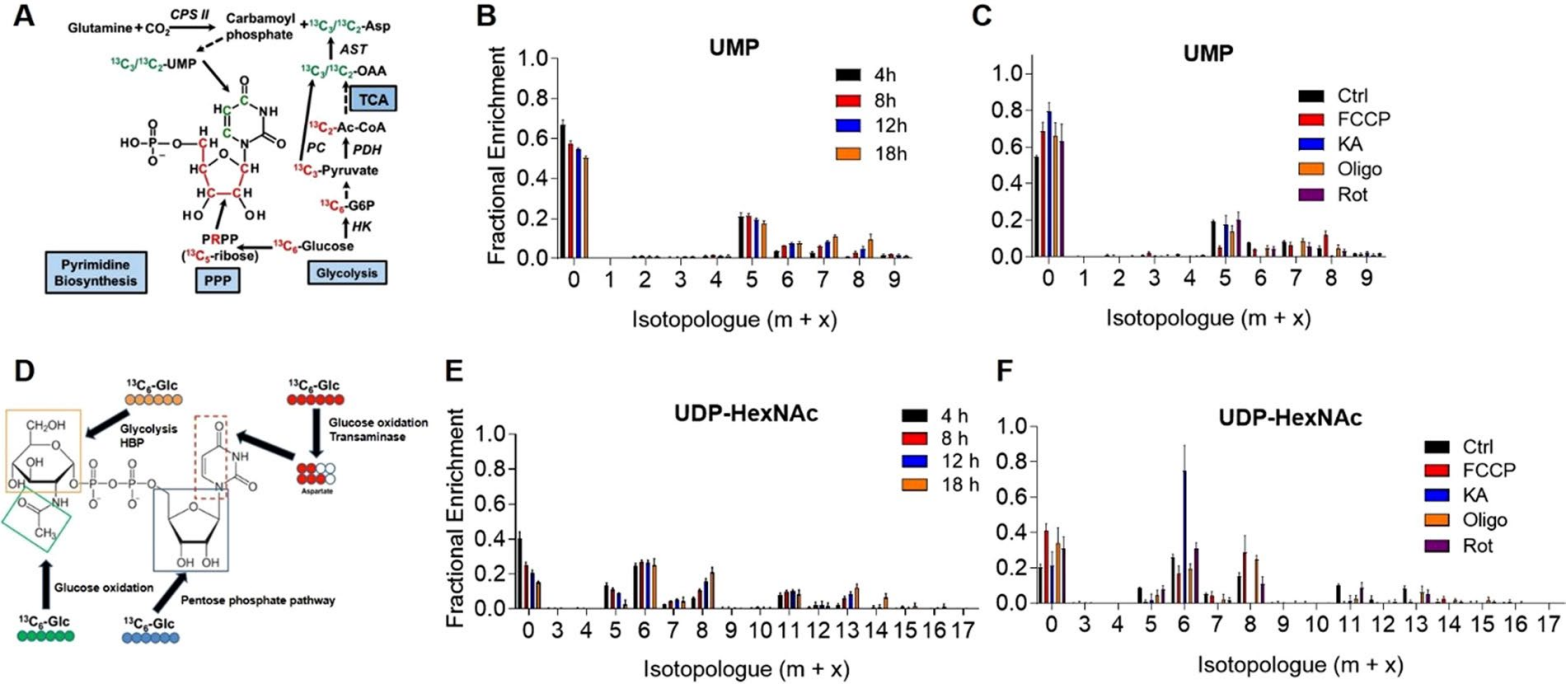
^13^C enrichment in biosynthetic pathway metabolites. Time course and pharmacological control ^13^C enrichment data: (**A**) Atom resolved map of pyrimidine (UMP) biosynthesis; (**B**) NRCMs were cultured in medium containing 25 mM ^13^C_6_-glucose for 4 h, 8 h, 12 h, or 18 h, followed by quenching, metabolite extraction, and SIRM analyses. Shown is the fractional enrichment plot of UMP; (**C**) NRCMs were incubated in 25 mM ^13^C_6_-glucose for 12 h with the indicated inhibitor (as in Figs 4–6), followed by SIRM analysis. Shown is the fractional enrichment plot of UMP; (**D**) Illustration of UDP-GlcNAc synthesis; (**E**) NRCMs were cultured in medium containing 25 mM ^13^C_6_-glucose for 4 h, 8 h, 12 h, or 18 h, followed by quenching, metabolite extraction, and SIRM analyses. Shown is the fractional enrichment plot of UDP-HexNAc (composed of both UDP-GlcNAc and UDP-GalNAc); and (**F**) NRCMs were incubated in 25 mM ^13^C_6_-glucose and the indicated inhibitor (as in Figs 4–6), followed by SIRM analysis. Shown is the fractional enrichment plot of UDP-HexNAc. n = 6 replicates per group, pooled from NRCMs isolated from three independent litters.

Biosynthetic pathway activity: In addition to catabolic pathways, SIRM provides information of how nucleotides, UDP-HexNAc (comprising UDP-GlcNAc and UDP-GalNAc), amino acids, and glycerophospholipids are built, which delineates anabolic pathway activity in cells or tissues^31,42,56^. As an example, we show in Fig. 7 fractional enrichment data in the pyrimidine, uridine monophosphate (UMP), and in the endproduct of the hexosamine biosynthetic pathway (HBP), UDP-HexNAc. The former is synthesized via the pentose phosphate pathway and from aspartate-derived carbons (Fig. 7A), and the latter is synthesized from carbons derived from not only F6P, but from acetyl CoA and UDP as well (Fig. 7D). In NRCMs, both UMP and UDP-HexNAc appear to remain in the dynamic phases of labeling through 12 h of ^13^C_6_-glucose provision (Fig. 7B,E).

The UMP labeling profile indicates that all inhibitors diminish incorporation of glucose-derived carbon into pyrimidines, with FCCP inhibiting glucose carbon incorporation into the ribose ring (m + 5). The labeling patterns of the m + 6, m + 7, and m + 8 isotopologues of UMP in FCCP-treated cells are consistent with that of the m + 2, m + 3, and m + 4 isotopologues of asparate, respectively (see Fig. 6). The pool size of UMP was modestly lower in FCCP-treated NRCMs (Supplementary Fig. 7).

A similar picture emerges with UDP-HexNAc. FCCP decreased glucose carbon incorporation into the m + 5, m + 6, m + 11, and m + 13 isotopologues of UDP-HexNAc, which largely represent carbons derived from UDP (m + 5), glycolysis-derived F6P (m + 6), UDP + F6P (m + 11), and UDP + F6P + glucose oxidation-derived acetyl CoA (m + 13) (Fig. 7F). These data indicate that mitochondrial uncoupling by FCCP diminishes pyrimidine synthesis by decreasing pentose phosphate pathway flux and that it may decrease UDP-HexNAc synthesis by diminishing carbon incorporation from UDP, F6P, and acetyl CoA.

Exposure of NRCMs to KA markedly diminished glucose-derived carbon incorporation into UMP (Fig. 7B), and it diminished UMP pool size (Supplementary Fig. 7A). Although KA did not affect ribose incorporation into UMP (m + 5), it markedly diminished incorporation from aspartate (see m + 6, m + 7, m + 8 isotopologues). Because KA markedly inhibited glucose oxidation and diminished the overall Krebs cycle intermediate pool size (Supplementary Fig. 6), these data indicate that KA inhibited pyrimidine biosynthesis by decreasing glucose oxidation and aspartate synthesis.

The ^13^C labeling patterns and pool size in UDP-HexNAc from KA-exposed NRCMs suggest that although inhibition of GAPDH drives F6P-derived carbon into the HBP, UDP-HexNAc biosynthesis is limited by availability of Krebs cycle-derived products. This conclusion is supported by the fact that KA increased ^13^C enrichment in the m + 6 isotopologue, yet diminished ^13^C incorporation into the ribose of UDP (m + 5 isotopologue) and into those isotopologues built from Krebs cycle-derived products (m + 7–m + 16 isotopologues). Furthermore, KA remarkably decreased UDP-HexNAc pool size. Oligomycin and rotenone treatment did not have large effects on UMP or UDP-HexNAc pool size or ^13^C enrichment. Collectively, these findings illustrate critical relationships between catabolic and anabolic pathways and show that mitochondrial uncoupling and glucose oxidation affect the rates of pyrimidine and UDP-HexNAc biosynthesis.

Summary: The findings of this study illustrate how to integrate SIRM with the use of pharmacological controls and complementary flux analyses, which collectively provide a more comprehensive view of metabolism. The pharmacological controls used here provide the experimenter the ability to appraise confidence in fractional enrichment data. An example shown here is α-ketoglutarate, the enrichment pattern of which led to its spectral reanalysis and exclusion from interpretation. The combined use of time course data and metabolic inhibitors and activators can be used to assess the relative difference in pathway activity. We provide evidence that, in NRCMs, glucose oxidation occurs at a higher rate than anaplerotic activity from pyruvate carboxylase or malic enzyme. Moreover, we provide tangible examples of how labeling at isotopic steady state does not provide an index of flux. Additionally, we found that, in NRCMs, alanine may be synthesized via an aspartate decarboxylase. The data also provide unique insights into how glycolysis, glucose oxidation, and mitochondrial uncoupling regulate ancillary biosynthetic pathway activity. Although these findings may be specific to the cultured NRCM system used here, they provide evidence that may extend to other *in vitro* or to *in vivo* systems.

## Materials and Methods

### Materials

DMEM growth medium containing l-glutamine, D-glucose, and sodium pyruvate was purchased from Sigma-Aldrich (St. Louis, MO, USA). Koningic acid was from Cayman Chemical (Ann Arbor, MI, USA). All other reagents were from Sigma-Aldrich Corp. (St. Louis, MO, USA), unless indicated otherwise.

### Rodent models & primary cell isolation

All procedures were approved by the University of Louisville Institutional Animal Care and Use Committee and were in accordance with NIH guidelines. The euthanasia procedures were consistent with the AVMA *Guidelines for the Euthanasia*. Neonatal rat cardiomyocytes (NRCMs) were isolated from 1- to 2-day-old Sprague-Dawley rats as previously described^57–59^. Briefly, neonatal rat hearts were excised, rinsed, and minced in calcium- and bicarbonate-free Hank’s balanced salt solution containing HEPES (pH 7.0). Cells were dissociated by stepwise trypsin digestion, after which the trypsin in the dissociated cell mixture was quenched with FBS and centrifuged at 180 *g* for 5 min. The pellet was then resuspended in warm DMEM (25 mM glucose, 4 mM glutamine, 1 mM sodium pyruvate) supplemented with 5% FBS, 1% penicillin/streptomycin, 0.1 mM BrdU, and 2 µg/mL vitamin B12 and preplated in 10 cm dishes for 1 h to allow for fibroblast adherence and removal. The non-adherent cardiomyocytes were then collected and plated at the desired cell density, as outlined below.

### Cell culture

NRCMs were plated into tissue culture treated 6-well dishes (1.7 × 10^6^ cells/well) or XF24 cell culture microplates (7.5 × 10^4^ cells/well) in DMEM (25 mM glucose, 4 mM glutamine, 1 mM sodium pyruvate) supplemented with 5% FBS, 1% penicillin/streptomycin, 0.1 mM BrdU, and 2 µg/mL vitamin B12. The following day (day 1), media was replaced with the same plating media. On day 4, culture media was changed to media lacking BrdU. On day 5, media was replaced to serum-free media for at least 18 h prior to experimental treatments. Following serum starvation, cells were treated with pharmacological and chemical reagents: FCCP − carbonyl cyanide 4-(trifluoromethoxy) phenylhydrazone (1 µM); KA – koningic acid (10 µM); Oligo – oligomycin (1 µM); Rot – rotenone (1 µM); or 2DG – 2-deoxyglucose (100 mM).

Cell viability before and following treatments were assessed via visual inspection under microscopy; there were no indications (vesicle formation, cell debris, etc.) of a change in cell viability following any the treatments. Assessment of total protein was used as an additional surrogate for changes in cell viability.

### Stable isotope tracing

NRCMs were incubated in 6-well plates for 5 min or 4–18 h in DMEM media (US Biological; Swampscott, MA, USA) containing 1 mM pyruvate, 4 mM glutamine, and 25 mM [^13^C_6_]-glucose (99% purity, microbiological and pyrogen tested; Cambridge Isotope Laboratories, Inc., Tewksbury, MA). Additionally, for compound/inhibitor experiments, FCCP, KA, Oligo, Rot, or 2DG were added to this [^13^C_6_]-glucose containing media. After the specified isotope labeling time point, cell reactions were quenched in cold acetonitrile, and extracted in acetonitrile:water:chloroform (v/v/v, 2:1.5:1), as described previously^31,32,60,61^, to obtain the polar, nonpolar, and insoluble proteinaceous fractions. The nonpolar (lipid) layer was collected, dried under a stream of nitrogen gas, and reconstituted in 0.1 ml of chloroform:methanol:butylated hydroxytoluene (2:1 + 1 mM) mixture and stored at −80 °C for future analysis. The polar fraction was lyophilized using a Freezone 2.5 L −84 °C benchtop freeze dryer (Labconco, Kansas City, CO, USA). The dried sample was reconstituted in 100 μl 20% acetonitrile and used for LC-MS analysis.

### LC-MS analysis

All samples were analyzed on a Thermo Q Exactive HF Hybrid Quadrupole-Orbitrap Mass Spectrometer with a Thermo DIONEX UltiMate 3000 HPLC system (Thermo Fisher Scientific, Inc., Germany). Separation was performed in parallel mode on a reversed phase (RP) Waters ACQUITY UPLC HSS T3 column (150 × 2.1 mm i.d., 1.8 µm, part number: 186003540) and a hydrophilic interaction chromatography (HILIC) Thermo Accucore HILIC column (100 × 3 mm i.d., 2.6 µm, part number: 17526–103030). The temperature of both columns was set to 40 °C. The HILIC column was operated as follows: mobile phase A was 10 mM ammonium acetate (pH adjusted to 3.25 with acetate) and mobile phase B was acetonitrile. The gradient was: 0 min, 100% B; 0 to 5 min, 100% B to 35% B; 5 to 12.7 min, 35% B; 12.7 to 12.8 min, 35% B to 95% B; 12.8 to 14.3 min, 95% B. The flow rate was set 0.3 mL/min. For the RP column, the mobile phase A was water with 0.1% formic acid and mobile phase B was 100% acetonitrile. The gradient was as follows: 0 min, 0% B; 0 to 5 min, 0% B; 5 to 6.1 min, 0 to 15% B; 6.1 to 10 min, 15 to 60% B; 10 to 12 min, 60% B; 12 to 14 min, 60% to 100% B; 14 to 14.1 min, 100% to 5% B; 14.1 to 16 min, 5% B. The flow rate was 0.4 mL/min.

The electrospray ionization probe was fixed at level C. The parameters for the probe were set as follows: sheath gas = 55 arbitrary units, auxiliary gas = 15 arbitrary units, sweep gas = 3 arbitrary units, spray voltage = 3.5 kV, capillary temperature = 320 °C, S-lens RF level = 65.0, auxiliary gas heater temperature = 450 °C. The method of mass spectrometer was set as follows: full scan range = 50 to 750 (m/z); resolving power R = 30,000 at m/z = 200, 10% valley; maximum injection time = 50 ms; automatic gain control (AGC) = 10^6^ ions for both positive and negative modes.

Unlabeled samples were analyzed in full MS scan and data-dependent MS/MS modes, while the labeled specimens were only analyzed in full MS mode. The LC methods and electrospray ionization conditions were set as follows: for full MS scan, scan range = 50–750 (m/z), R = 30,000 at m/z = 200, 10% valley, maximum injection time = 50 ms, automatic gain control (AGC) = 10^6^ ions; for data dependent MS/MS scan, R = 15,000, maximum injection time = 100 ms, automatic gain control (AGC) = 5 × 10^4^, loop count = 6, isolation window = 1.3 m/z, dynamic exclusion time = 1.2 s, the collision energy was set 10, 20, 40, 60 and 150 eV, respectively.

### Stable isotope data analyses

Full MS.raw files were first converted to.mzML format with msConvert tool, a part of an open-source ProteoWizard suite, described in detail by Adusumilli and Mallick^62^. Isotopologue peak deconvolution and assignments were performed using El-MAVEN. Peaks were assigned using a metabolite library first generated and verified using full scan MS and MS/MS spectra of unlabeled samples, as described previously^33,61^. The library contained metabolite names and corresponding molecular formulae used for generation of theoretical m/z values for all possible isotopologues, and retention times for each entry. The El-MAVEN parameters for compound library matching were as follows: EIC Extraction Window ± 7 ppm; Match Retention Time ± 0.60 min. For ^13^C isotopologue peak detection, the software criteria were set as follows: Minimum Isotope-parent correlation 0.5; Isotope is within 7 scans of parent; Abundance threshold 1.0; Maximum Error To Natural Abundance 100%. All assignments were visually inspected and compared to unlabeled samples for reference. Any peak groups assigned in error, e.g. not present or having different retention time than in the unlabeled samples, were deleted and correct peak assignments were added manually. Finally, the peak list with corresponding abundances was exported to a comma-separated (CSV) file and uploaded to the Polly workflow to perform natural abundance correction and to calculate total pool size for each metabolite (by summing peak areas of each detected isotopologue) using Polly Isocorrect. Finally, the data were analyzed and plotted with GraphPad Prism 8.0 (GraphPad Software, San Diego, Ca, USA).

### Radiolabeled glucose utilization assay

Glucose utilization was determined using [5-^3^H]-glucose^63–65^. Briefly, NRCMs were cultured in 6-well dishes and culture media was replaced with 2 ml of fresh, serum-free culture media with the addition of 2 µCi/ml [5-^3^H]-glucose (Moravek Biochemicals, Brea, CA, USA). Following incubation for 3 h, 100 µl of media was collected and added to 100 µl of 0.2 M HCl in a microcentrifuge tube. This tube, with the tube cap removed, was placed in a scintillation vial containing 500 µl of dH_2_O to allow for evaporation diffusion of [^3^H]_2_O in the microcentrifuge tubes into the scintillation vials. To account for incomplete equilibration of [^3^H]_2_O and background, known amounts (µCi) of [5-^3^H]-glucose and [^3^H]_2_O (Moravek Biochemicals) were placed into microcentrifuge tubes, and these were placed into separate scintillation vials containing 500 µl dH_2_O. After incubation at 37 °C for 72 h, the microcentrifuge tube was removed from the vial, 10 ml of scintillation fluid was added, and scintillation counting was performed using a Tri-Carb 2900TR Liquid Scintillation Analyzer (Packard Bioscience Company, Meriden, CT, USA). Glucose utilization was then calculated as reported by Ashcroft *et al*.^63^, with considerations for the specific activity of [5-^3^H]-glucose, incomplete equilibration and background, dilution of [5-^3^H]- to unlabeled-glucose, and scintillation counter efficiency. To normalize glucose utilization to total protein, the cells were washed with PBS (to remove adherent radioactivity) and then lysed in common lysis buffer. Protein concentration was measured using the Lowry assay kit (Bio-Rad Laboratories).

### Glucose uptake measurements

Glucose uptake was estimated by uptake of ^3^H-2-deoxyglucose (2DG)^59^. For this, NRCMs in 6-well plates were exposed to 2 µCi/ml ^3^H-2-DG in the presence or absence of 100 mM unlabeled 2-DG, 10 µM KA, or 1 µM FCCP for 3 h. The cells were then washed 6 times with ice-cold phosphate-buffered saline to remove adherent radioactivity, and lysed in 100 µl of lysis buffer (20 mM Hepes, pH 7.0, 1 mM EDTA, 1% Nonidet P40 and 0.1% SDS). After centrifugation at 13,000 *g* for 10 min, radioactivity in the supernatant was measured by scintillation counting. The radioactivity (in counts per minute; CPM) was normalized to total protein content.

### Extracellular flux analysis of cellular energetics

To measure cellular energetics in intact cardiomyocytes, the Seahorse Bioscience XF24 extracellular flux analyzer was used as previously described^47^. For this, 75,000 NRCMs were plated into each well and cultured as described above. An hour before each experiment, the media was replaced with 675 µl of assay media, i.e., unbuffered DMEM containing glucose (25 mM), glutamine (4 mM), and pyruvate (1 mM), pH 7.4. Following 1 h in a 37 °C, CO_2_-free incubator, the cells were placed in the instrument for analysis. Basal oxygen consumption rates (OCR) and extracellular acidification rates (ECAR) were measured using a programmed protocol: 3 cycles of 2 min mix, 2 min wait, and 3 min measure. The OCR and ECAR values were normalized to the total amount of protein in each well.

### Statistical analysis

All values are mean ± S.D. Comparisons were made to the relevant control group, and statistical tests were performed using one-way analysis of variance (ANOVA) with Dunnett’s correction for multiple comparisons. The null hypothesis was rejected when *p* < 0.05.

## Supplementary information

Data supplement

## Data Availability

Raw data are available upon request.
