# Supplementary material for: Integration of flux measurements and pharmacological controls to optimize stable isotope-resolved metabolomics workflows and interpretation
Source: Sci Rep. 2019 Sep 23;9:13705. doi: 10.1038/s41598-019-50183-3 (PMC6757038; doi:10.1038/s41598-019-50183-3)
Supplement: Supplementary file 1 — Data supplement [file 41598_2019_50183_MOESM1_ESM.pdf]

DATA SUPPLEMENT

Integration of flux measurements and pharmacological controls to optimize stable isotope-resolved metabolomics workflows and interpretation

#Pawel K. Lorkiewicz[1,2], #Andrew A. Gibb[1,4], Benjamin R. Rood[1], Liqing He[2], Yuting Zheng[1], Brian F. Clem[3], Xiang Zhang[2], and *Bradford G. Hill[1]

#These authors contributed equally to this work.

[1]Department of Medicine, Division of Environmental Medicine, Christina Lee Brown Envirome Institute, Diabetes and Obesity Center, Envirome Institute, [2]Department of Chemistry, Center for Regulatory and Environmental Analytical Metabolomics, [3]Department of Biochemistry and Molecular Genetics, University of Louisville, Louisville, KY and [4]Lewis Katz School of Medicine, Temple University, Philadelphia, PA

Supplementary Fig. 1

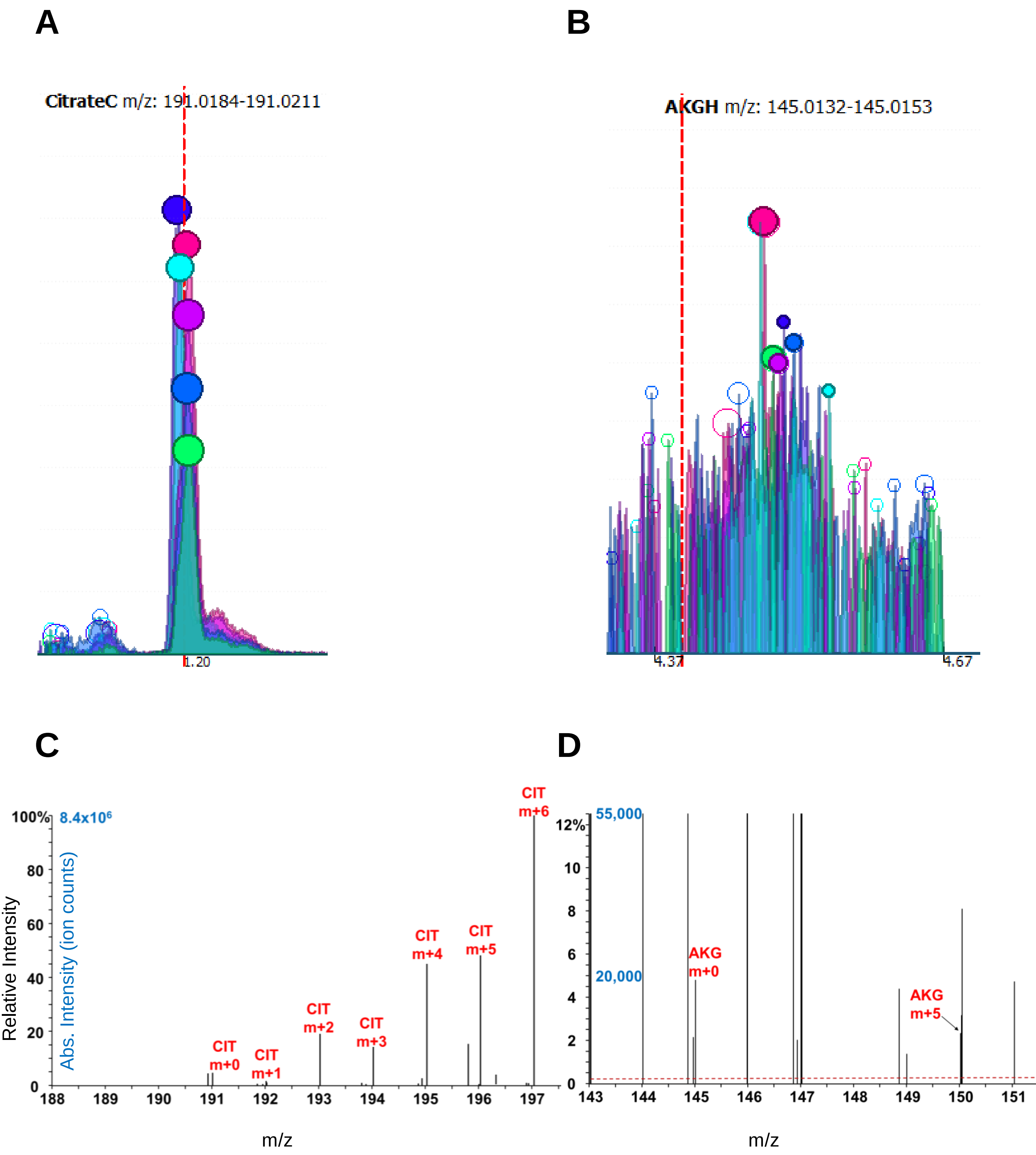

Supplemental Fig. 2

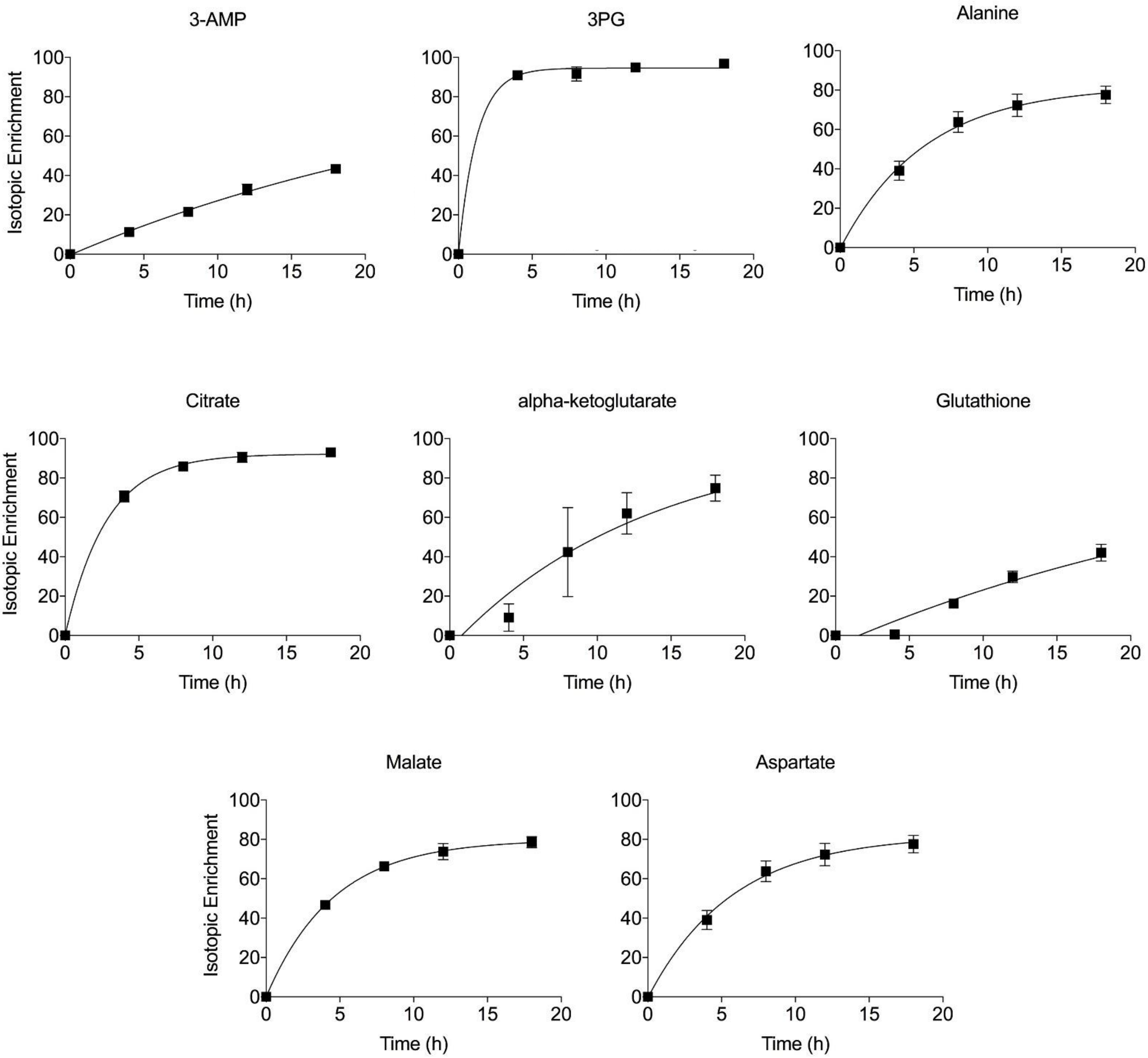

Supplemental Fig. 3

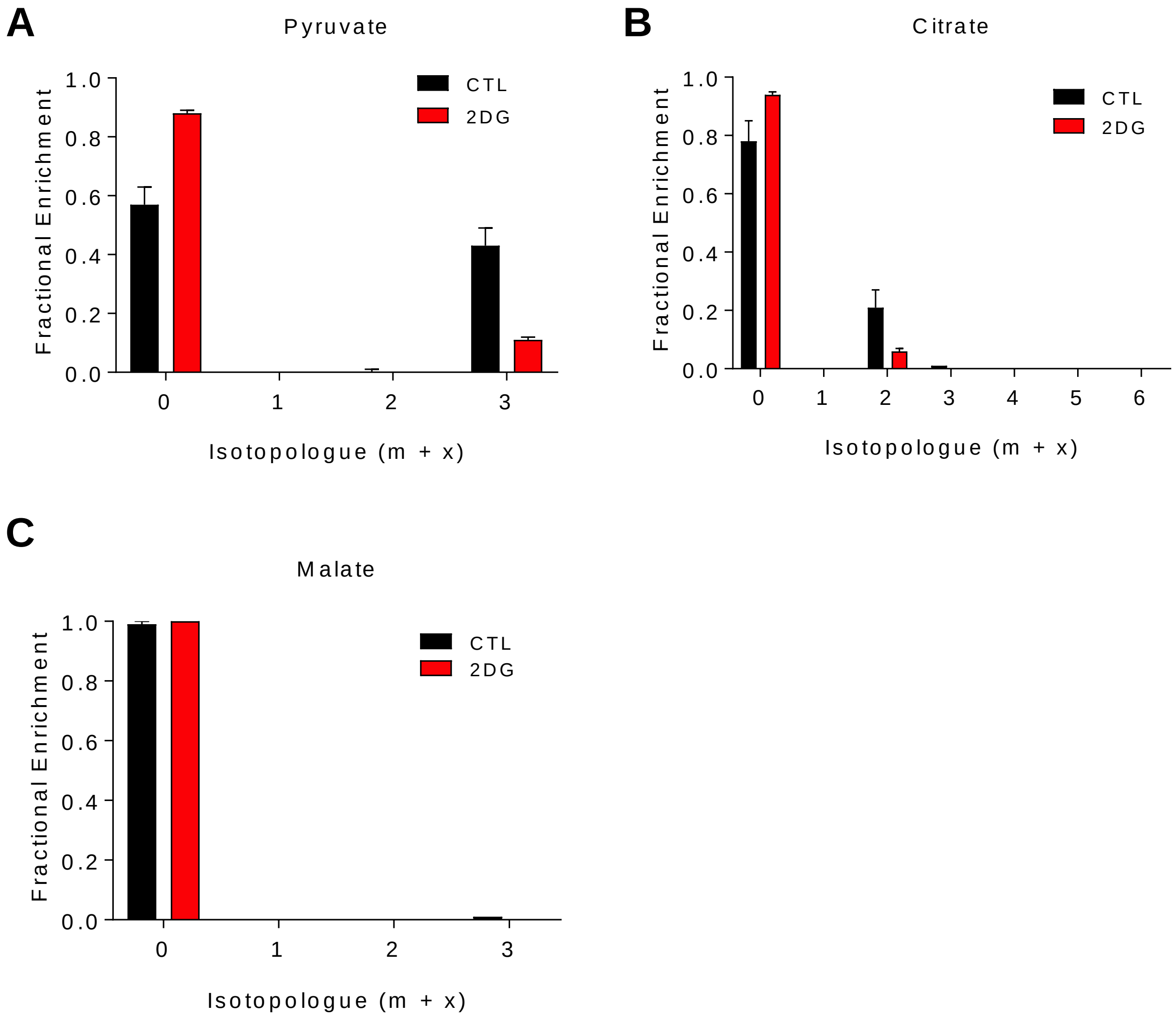

Supplemental Fig. 4

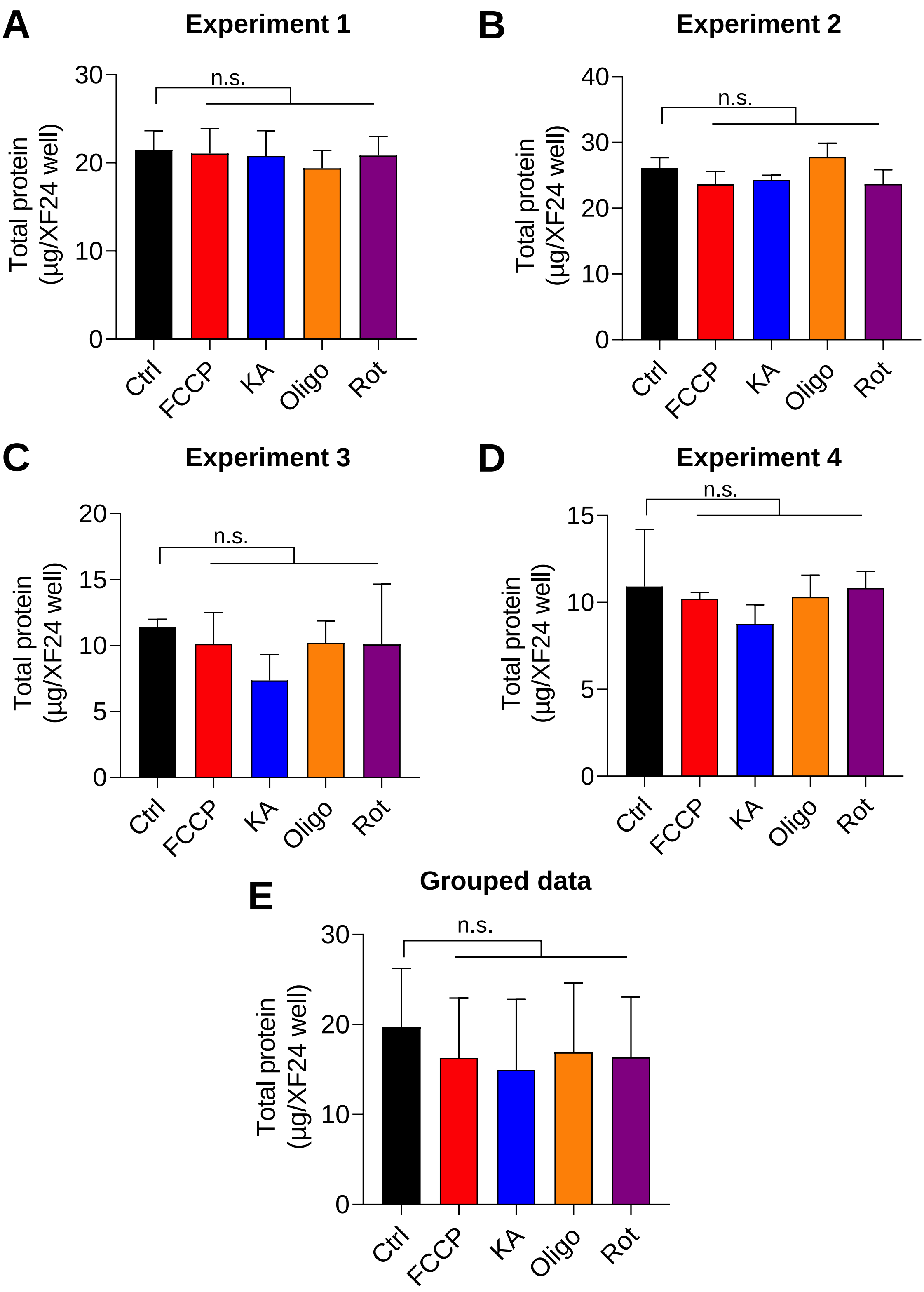

Supplementary Fig. 5

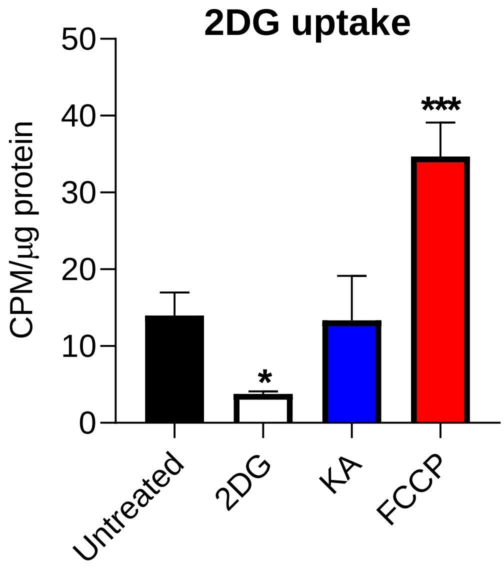

Supplementary Fig. 6

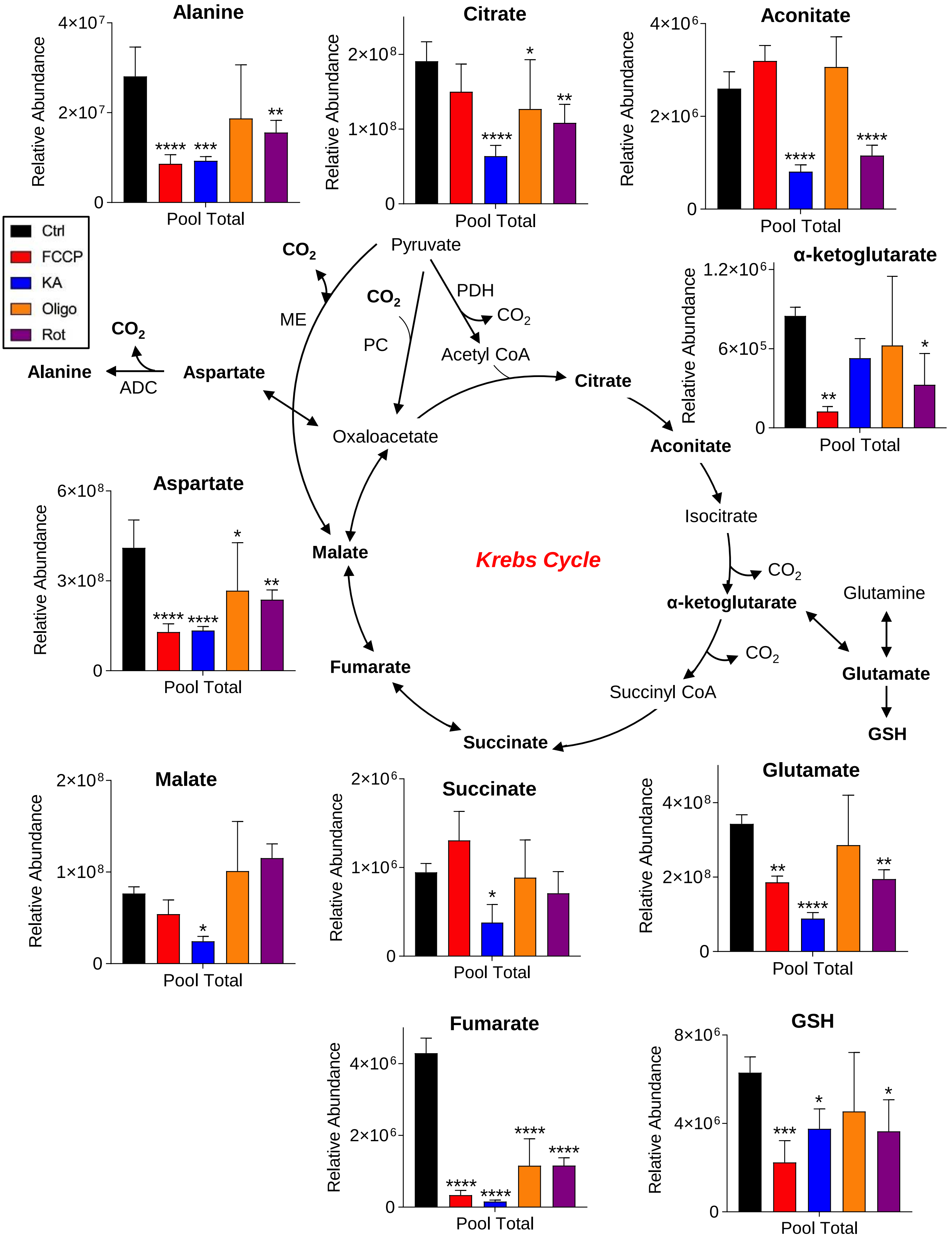

Supplementary Fig. 7

A

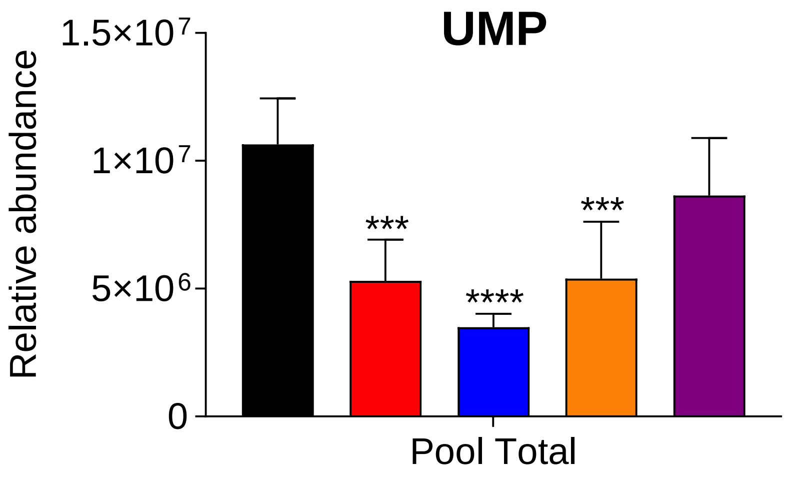

B

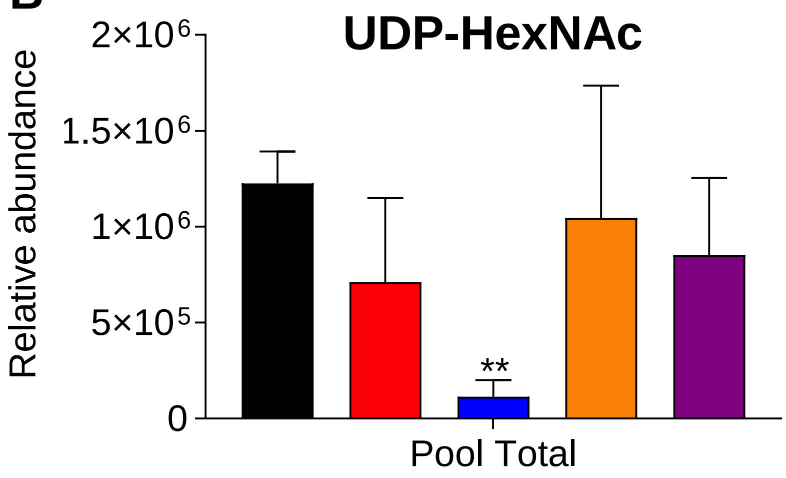

SUPPLEMENTARY FIGURE LEGENDS

Supplementary Fig. 1: Example chromatograms and corresponding mass spectra of high and low abundance ^{13}C-labeled metabolites. Neonatal rat cardiomyocytes were incubated for 12 h in medium containing 25 mM $^{13}C_6$-glucose and 1 µM FCCP. Intracellular metabolites were then extracted and subjected to LC/MS. Shown are representative chromatographic peaks of (**A**) total citrate (CIT) and (**B**) α-ketoglutarate (AKG) in six overlaid replicates. Corresponding fragments of full MS spectra (before natural abundance correction) are shown for citrate (**C**) and α-ketoglutarate (**D**). The red dotted lines in panels A and B represent expected retention times. The dotted line in panel D represents the noise level threshold.

Supplementary Fig. 2: Time course of metabolite labeling. Neonatal rat cardiomyocytes were cultured in medium containing 25 mM $^{13}C_6$-glucose for 4–18 h, followed by LC/MS analysis. Shown are metabolites indicative of catabolic (e.g., 3PG, citrate, α-ketoglutarate, and malate) and anabolic (3-AMP, alanine, glutathione, aspartate) pathways. n=6 replicates per group, pooled from NRCMs isolated from three independent litters.

Supplementary Fig. 3: Use of acute labeling strategies to differentiate relative metabolic pathway activity. Neonatal rat cardiomyocytes were cultured in medium containing 25 mM $^{13}C_6$-glucose for 5 min in the presence or absence of 100 mM 2-deoxyglucose (2DG). Shown are pyruvate (panel **A**), citrate (panel **B**), and malate (panel **C**). The 2DG was used as a control to ensure confidence in accurate assignment. n=6 replicates per group, pooled from NRCMs isolated from three independent litters.

Supplementary Fig. 4: Protein content is unchanged by pharmacological agents. Protein content of neonatal rat cardiomyocytes treated with FCCP, KA, Oligo, or Rot: (**A-D**) Total protein per well from XF24 plates following treatment with pharmacological agents for 12 h. n = 3-7 technical replicates per group. (**E**) Aggregate data integrating total protein data from panels A-D. n = 4 independent experiments.

Supplementary Fig. 5: Effects of KA and FCCP on glucose uptake. Neonatal rat cardiomyocytes were cultured in DMEM containing 25 mM glucose and 2 µCi/ml ^{3}H-2-deoxyglucose (2DG) for 3 h in the presence or absence of 100 mM unlabeled 2DG, 10 µM KA, or 1 µM FCCP. Uptake of radiolabeled 2DG was measured in cell lysates by scintillation counting and normalized to cell protein. n=3 replicates per group, pooled from NRCMs isolated from two independent litters. *$p<0.05$, ***$p<0.005$ compared with control group.

Supplementary Fig. 6: Relative pool size of Krebs cycle intermediates. Neonatal rat cardiomyocytes were cultured in medium containing 25 mM $^{13}C_6$-glucose for 12 h in the presence or absence of the indicated pharmacological agents (as in Figs. 4, 5, and 6). For each metabolite, relative abundances were calculated by summing the peak intensities from each isotopologue. n=4-6 replicates per group, pooled from NRCMs isolated from three independent litters. *$p<0.05$, **$p<0.01$, ***$p<0.005$, ****$p<0.0001$ vs. Ctrl.

Supplementary Fig. 7: Relative pool size of UMP and UDP-HexNAc. Neonatal rat cardiomyocytes were cultured in medium containing 25 mM $^{13}C_6$-glucose for 12 h in the presence or absence of the indicated pharmacological agents (as in Figs. 4, 5, and 6). For each metabolite, relative abundances were calculated by summing the peak intensities from each isotopologue. n=6 replicates per group, pooled from NRCMs isolated from three independent litters. **$p<0.01$, ***$p<0.005$, ****$p<0.0001$ vs. Ctrl.
